# The structure of FMNL2–Cdc42 yields insights into the mechanism of lamellipodia and filopodia formation

**DOI:** 10.1038/ncomms8088

**Published:** 2015-05-12

**Authors:** Sonja Kühn, Constanze Erdmann, Frieda Kage, Jennifer Block, Lisa Schwenkmezger, Anika Steffen, Klemens Rottner, Matthias Geyer

**Affiliations:** 1 Center of Advanced European Studies and Research, Group Physical Biochemistry, Ludwig-Erhard-Allee 2, Bonn 53175, Germany; 2 Department of Physical Biochemistry, Max Planck Institute of Molecular Physiology, Otto-Hahn-Strasse 11, Dortmund 44227, Germany; 3 Institute of Genetics, Actin Dynamics and Motility Unit, University of Bonn, Karlrobert-Kreiten-Strasse 13, Bonn 53115, Germany; 4 Division of Molecular Cell Biology, Zoological Institute, Technical University Braunschweig, Spielmannstrasse 7, Braunschweig 38106, Germany; 5 Helmholtz Centre for Infection Research, Department of Cell Biology, Inhoffenstrasse 7, Braunschweig 38124, Germany; 6 Institute of Innate Immunity, Department of Structural Immunology, Sigmund-Freud-Str. 25, Bonn 53127, Germany

## Abstract

Formins are actin polymerization factors that elongate unbranched actin filaments at the barbed end. Rho family GTPases activate Diaphanous-related formins through the relief of an autoregulatory interaction. The crystal structures of the N-terminal domains of human FMNL1 and FMNL2 in complex with active Cdc42 show that Cdc42 mediates contacts with all five armadillo repeats of the formin with specific interactions formed by the Rho-GTPase insert helix. Mutation of three residues within Rac1 results in a gain-of-function mutation for FMNL2 binding and reconstitution of the Cdc42 phenotype *in vivo*. Dimerization of FMNL1 through a parallel coiled coil segment leads to formation of an umbrella-shaped structure that—together with Cdc42—spans more than 15 nm in diameter. The two interacting FMNL–Cdc42 heterodimers expose six membrane interaction motifs on a convex protein surface, the assembly of which may facilitate actin filament elongation at the leading edge of lamellipodia and filopodia.

## Abstract (web summary)

FMNL formins polymerize actin filaments to generate cellular protrusions such as lamellipodia and filopodia at the leading edge of a cell. Here the authors provide detailed mechanistic insights into the formation of actin-based protrusions through GTPase dependent activation and membrane localization of FMNL1 and FMNL2.

## Graphical abstract

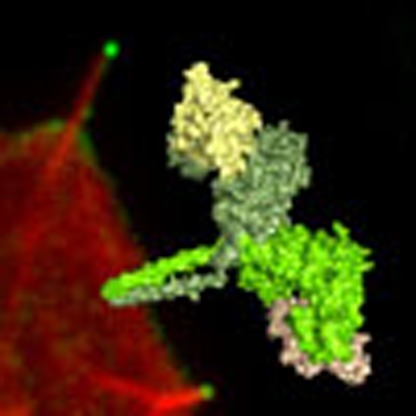

The actin cytoskeleton is a highly dynamic structure that regulates cell shape and motility. The assembly of globular actin molecules into actin filaments (F-actin) is spatially and temporally controlled by nucleation and elongation factors. Among these, the multidomain proteins of the formin family regulate a variety of distinct F-actin structures in vertebrates, such as stress fibres, filopodia, lamellipodia or cell adhesions^1^,^2^,^3^. Formins thus influence a large number of major cellular processes like cell migration, polarity and cell cycle progression. The 15 mammalian formins that are defined by the presence of formin homology (FH) domains cluster into 8 different families based on their domain architectures^4^.

The actin polymerization activity of formins is mediated by the FH2 domain that resembles an anti-parallel, torus-shaped dimer, which remains associated with the F-actin barbed end on elongation^5^,^6^. The FH2 domain is preceded by a proline-rich FH1 domain that interacts with profilin-actin and accelerates the elongation rate of actin filaments^7^,^8^. For some formins, the region C-terminal to the FH2 domain may additionally contribute to actin polymerization kinetics^9^,^10^,^11^. Besides regulating elongation, formins possess actin nucleation^12^,^13^,^14^, filament bundling^15^ and presumably severing^11^ activity. The activity of some formins is regulated by an N- to C-terminal intramolecular interaction that renders the protein inactive, giving rise to the classification as Diaphanous-related formins (DRFs). In DRFs, the N-terminal regulatory domains comprise a GTPase-binding domain (GBD) followed by an FH3 domain that interacts with the C-terminal Diaphanous-autoregulatory domain (DAD). The inhibition is released on binding of an active Rho-GTPase to the formin GBD^16^,^17^,^18^. With the exception of FHOD, all mammalian DRFs also harbour a dimerization element C-terminal to the FH3 domain, constituting a second seed for the overall assembly of these proteins into multimers.

Rho family GTPases cycle between an inactive, GDP-bound state and an active, GTP-bound state^19^. In the active state, they have been shown to regulate cellular signalling by binding to a large variety of downstream effectors linking extracellular stimuli to actin cytoskeleton rearrangements^20^. Activation of the autoinhibited formin is supposed to occur on displacement of the autoregulatory DAD by the GTPase^17^,^18^,^21^. However, additional signals such as phosphorylation of the DAD or other DAD-interacting factors may also stabilize the active conformation of the DRF^22^.

The mammalian formin-like proteins constitute one family of DRFs, which include the large, multidomain proteins FMNL1, FMNL2 and FMNL3 (also known as FRL1 or formin-related gene in leucocytes, FRL3/FHOD2 and FRL2/FHOD3, respectively)^23^. An actin filament polymerization and bundling activity of FMNL formins have been described^24^,^25^,^26^,^27^,^28^; in addition, FMNL1 appears to sever actin filaments^24^. The misregulation of FMNL proteins has been shown to cause various tumours, such as chronic lymphatic leukaemia or non-Hodgkin's lymphoma^29^,^30^, but also colorectal carcinoma^31^ and breast carcinoma^32^. In agreement with this, the macrophage-enriched FMNL1 has been reported to be involved in cell adhesion, migration and survival^23^. FMNL1 has furthermore been reported to be an essential regulator of centrosome polarity^33^, to exhibit crucial functions in the cytotoxic activation of T cells^33^ and to be required for Fcγ receptor-induced phagocytosis^34^. FMNL2 contributes to the formation of protrusive actin structures such as lamellipodia and filopodia at the leading edge of migrating cells^28^,^35^. Consistently, overexpression of this protein appears to cause metastasis of tumour cells^31^,^36^. Furthermore, depletion of FMNL2 has been implicated in developmental disorders like mental retardation, premature onset of puberty and limb abnormalities^37^.

To date, it remains unclear how FMNL formins are activated and how the specificity of GTPases for formin effectors is determined. Several Rho GTPases such as RhoC, Rac1 and Cdc42 have been implicated in the activation of FMNL1 and FMNL2 (refs 23, 28, 32, 33, 34, 38, 39, 40). Aside from interacting with Rho GTPases, membrane targeting mechanisms emerged as an important feature of formins, as the actin-based protrusions are confined to distinct cellular compartments. We have previously shown that Cdc42 recruits full-length FMNL2 into lamellipodia and filopodia^28^. Here we uncover the structural basis of the specificity of FMNL1 and FMNL2 binding towards Cdc42, its membrane association, as well as the dimerization properties of the formin. We find that the insert helix of Cdc42 contributes essential interactions to the formin effector and that the protein complex forms a convex membrane interaction site to promote filopodia and lamellipodia outgrowth.

## Results

### Overall structure of the Cdc42–FMNL2_N_ complex

**Figure 1:**
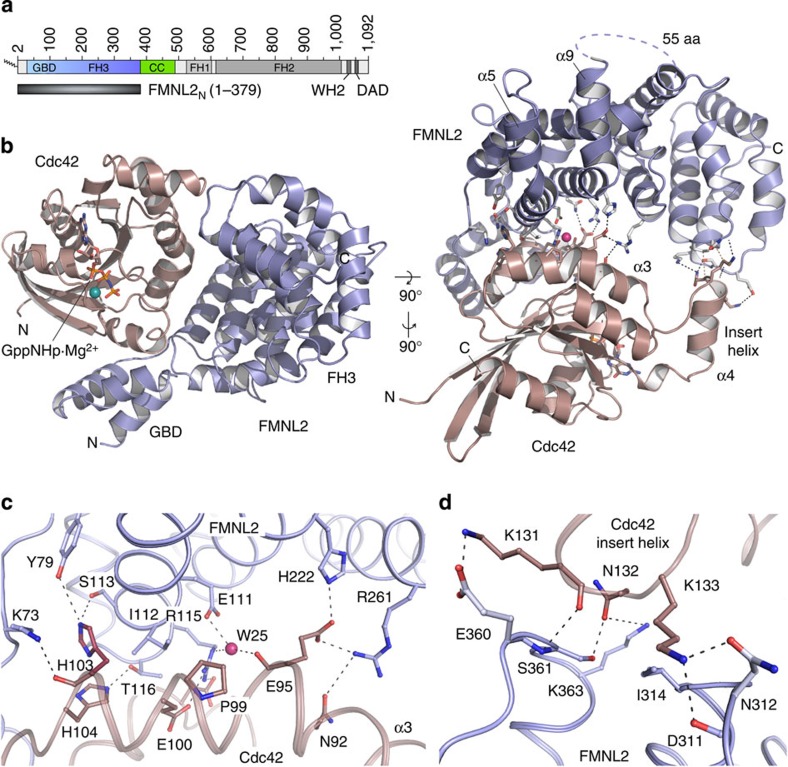
Structural basis of FMNL2 binding to Cdc42. (**a**) Domain architecture of human FMNL2. A protein construct encompassing the N-terminal GBD and FH3 domains was used for complex crystallization with human Cdc42. (**b**) Overall structure of the FMNL2_N_–Cdc42
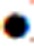
GppNHp complex shown in cartoon representation. The full GBD-FH3 domain entity is involved in binding to activated Cdc42. A network of polar interactions is formed between the armadillo repeats of FMNL2 and helices α3 and α4 (the 'insert helix') of Cdc42. (**c**) Close up of polar interactions formed between residues on helix α3 of Cdc42 and the first three repeats of the FMNL2 FH3 domain. Particularly E95 of Cdc42 forms salt bridges with H222 and R261 of the second and third armadillo repeat of FMNL2, respectively, and a water-mediated contact with E111 of the first heat repeat. (**d**) Close up of the interaction network between K131, N132 and K133 of the insert helix of Cdc42 and residues of the fourth and fifth armadillo repeat in FMNL2.

To explore the activating complex between Cdc42 and FMNL2, we solved the X-ray crystal structure of the GBD-FH3 domains of human FMNL2 (amino acids (aa) 1–379, termed FMNL2_N_) with Cdc42 (1–179) bound to the non-hydrolyzing nucleotide analogue GppNHp (Fig. 1a,b). The structure was determined to 2.5 Å resolution; the asymmetric unit of the crystal contained one heterodimer and the structure model was refined to an *R*_work_ of 19.4% and *R*_free_ of 25.3% with excellent stereochemistry (Table 1). Compared with the published structure of mDia1_N_ bound to Cdc42 (ref. 21), the FMNL2_N_–Cdc42
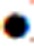
GppNHp structure shows remarkable differences in the interaction of the GTPase with the armadillo repeat helices of the formin. The buried molecular surface area of 3,034 Å^2^ counting both molecules FMNL2_N_ and Cdc42 is 51% larger than that of mDia1_N_–Cdc42. This increase in interaction surface is mostly due to additional contacts formed between the Cdc42 insert helix and the fourth and fifth armadillo repeat of the FH3 domain.

The FH3 domain of FMNL2 is composed of 14 canonical helices that assemble into 5 armadillo repeats with the first 2 helices resembling a heat repeat fold. This leads to formation of a combined surface of five long helixes at the concave site of the curved armadillo repeat structure. Two non-canonical helices (α5 and α9) are located before the long helices of the first two repeats (Fig. 1b). Both helices are kinked by ∼60° from the concave surface of the armadillo repeat. A loop sequence of 55 residues (150–204) that is not resolved in the crystal structure precedes the second helix of this non-canonical element. The N-terminal three helices (33–69) of the GBD are arranged similarly as seen in the mDia1_N_–RhoC complex^17^ with the connecting linker sequence (70–76) to the armadillo repeat domain being fully resolved in our crystal structure. Multiple hydrophobic and polar contacts are formed between the switch regions of Cdc42 and the armadillo repeats of FMNL2. Particularly, V36 and F37 of switch I and L67 and L70 of switch II of the GTPase contribute with hydrophobic interactions to the GBD of the formin (Supplementary Fig. 1a). Salt bridges and polar interactions are formed between E62, D63, D65 and R66 of switch II that reach out up to the second armadillo repeat of the FMNL2 effector domain (Supplementary Fig. 1b).

### Cdc42 interacts with all armadillo repeats of FMNL2

**Figure 2:**
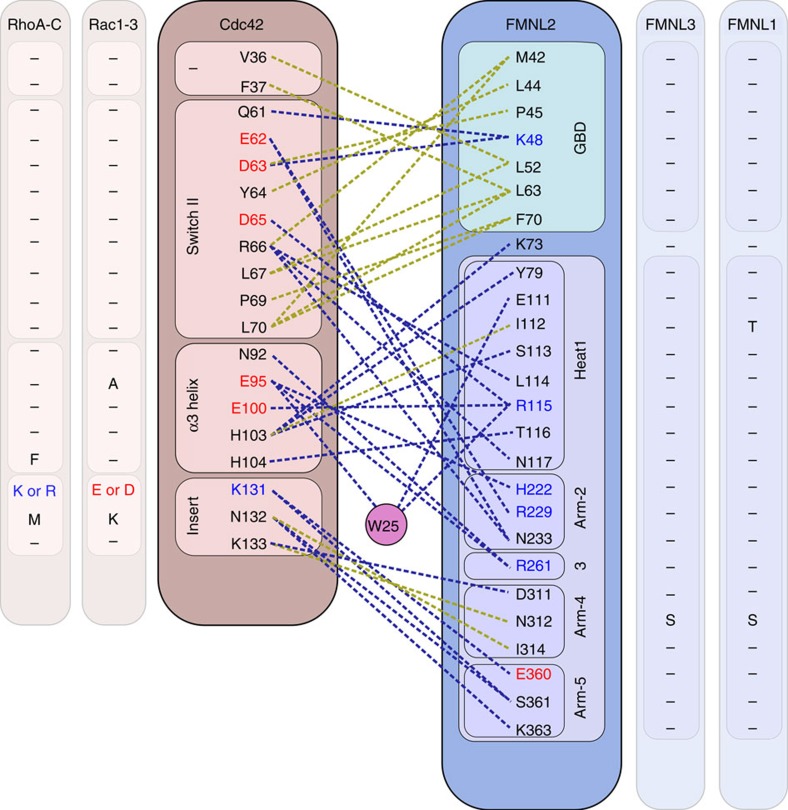
Interaction scheme between Cdc42 and FMNL2. Interactions between Cdc42 and FMNL2 involve the entire GBD-FH3 domain assembly up to the fifth armadillo repeat of the formin. Hydrophobic interactions are shown in ochre, polar interactions including hydrogen bonds and salt bridges are shown in blue dashed lines. Sequence variations of residues in the interaction network of GTPases RhoA to RhoC and Rac1 to Rac3 as well as within the FMNL formin family are shown at the sides, respectively. While the switch regions are fully conserved, changes in the interface occur in the insert helix and partly in helix α3.

Strikingly, residues of all five armadillo repeats of the FMNL2 FH3 domain participate in Cdc42 binding and contribute to the specificity of the interaction. A network of polar hydrogen bonds, whose alternating charges resemble a kind of electrostatic zipper, recurs throughout the beginning of the long armadillo repeat helices of FMNL2 (Fig. 1c,d). These residues mediate interactions with the switch II helix of Cdc42, the characteristic α3-helix and the Rho-GTPase-specific insert helix of Cdc42. Rho GTPases contain an insert of about 12 residues that forms the exposed 'insert helix' α4 and distinguishes this GTPase family from all other Ras-like GTPases (Supplementary Fig. 2). A superimposition of the FH3 domains of FMNL2 and mDia1 shows that helices α3 and α4 of Cdc42 are coming significantly closer to the armadillo repeat structure of FMNL2 compared with the mDia1_N_–Cdc42 complex structure (Supplementary Fig. 3a), yielding additional interactions between the two subunits. An interaction scheme displaying the contacts between FMNL2 and Cdc42 within a distance shell of 3.7 Å is shown in Fig. 2.

Cdc42, Rac1 to -3 and RhoA to -C all contain a conserved proline residue in the α3-helix that aligns to position 99 of Cdc42 (Supplementary Fig. 2b). The presence of this proline within a helix leads to formation of a characteristic bulge four residues upstream, likely due to the missing ability to form a hydrogen bond within the helical backbone (Fig. 1c). This results in exposure of E95 in Cdc42, which is characteristic for Rho GTPases and observed also in the absence of an effector protein. On complex formation with FMNL2, the carboxyl groups of E95 mediate salt bridges with H222 and R261 of the formin, thus coordinating two armadillo repeats of the effector protein (Fig. 1c). This specific interaction shown in a stereo display of the electron density map (Supplementary Fig. 3b) is only possible because of the exposed position of the glutamate in the helical bulge of the GTPase.

Second, a KNK motif at the tip of the Rho-GTPase-specific insert helix participates in the interaction with FMNL2. Polar interactions and a salt bridge are formed between the amino groups of the two lysines K133 and K131 in Cdc42 with acidic residues E311 and D360 of the fourth and fifth armadillo repeat of FMNL2, respectively. The interaction is sustained by a hydrogen bond between N132 and S361 (Fig. 1d). To our knowledge, this is the first observation of an effector-specific interaction of the Rho-GTPase insert helix with an effector protein. While residues in the switch I and switch II regions are rather well conserved in the Rho-GTPase family, compared, for example, with the Ras superfamily, it is the insert helix that exhibits higher sequence variability and can thus contribute to the specificity of the GTPase–effector interaction (Fig. 1d).

The interactions of Cdc42 with the N-terminal GBD of FMNL2 are largely of hydrophobic nature and overall similar to the binding observed in the mDia1_N_–RhoC complex formation^17^. A previously unseen interaction is formed between K73 in the linker connecting the GBD with the armadillo repeats of FMNL2 with the backbone oxygen of H103 in Cdc42 (Fig. 1c). The three helices of the GBD appear flexible and not well coordinated in the absence of the GTPase, a feature that classifies the GBD as a subdomain rather than a full domain entity.

### A Rac1 gain-of-function mutation for FMNL2 binding

To examine the contribution of the Rho-GTPase-specific insert helix for FMNL2 binding, we aimed at generating a Rac1 gain-of-function mutation for the interaction with the formin effector protein. We analysed the binding of Cdc42
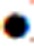
GppNHp to FMNL2_N_ by isothermal titration calorimetry (ITC) and determined a dissociation constant of 1.9 μM, whereas no interaction was seen for either Rac1 or RhoA binding (Table 2 and ref. 28). Proteomic analyses identified a phosphorylation site for S171 (ref. 41), which is in the centre of the flexible loop preceding helix α9 (Supplementary Fig. 4) and not resolved in the crystal structure. We introduced two aspartates at the site of S171 as a mimic of a negatively charged phosphorylation group. Using this S171DD mutant protein, the affinity of Cdc42
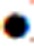
GppNHp for FMNL2_N_ was further improved to 0.79 μM, possibly due to increased protein stability and solubility in course of the ITC measurement.

**Figure 3:**
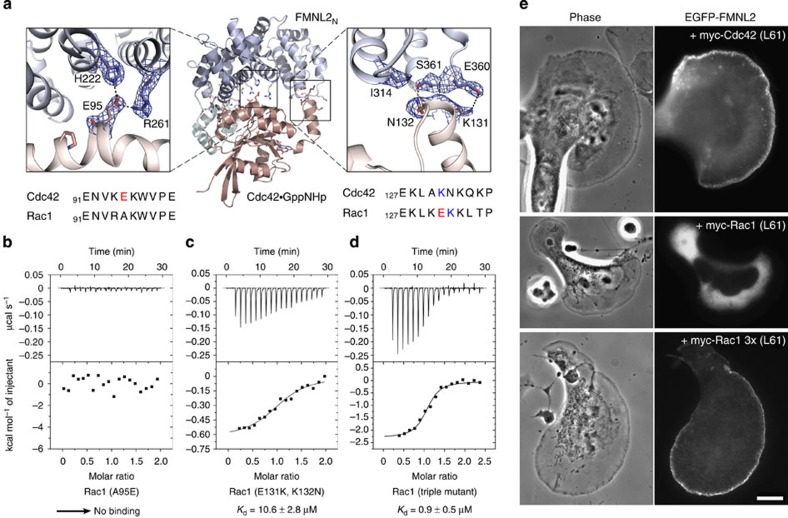
A triple mutation transforms Rac1 into a FMNL2 activator. (**a**) Display of the GTPase-specific interaction sites in the FMNL2_N_–Cdc42 interface. The electron density map is shown at 1σ. The sequences of Cdc42 and Rac1 in the α3-helix and the Rho insert helix α4 are shown below the structure figure. (**b–d**) Isothermal titration calorimetry measurements of Rac1 mutant binding to FMNL2_N_. No binding was detected for the single mutant (A95E) (**b**), whereas the double mutation (E131K,K132N) (**c**) and the combination of both sites to a triple mutation (**d**) increased binding of Rac1 to 10.6 and 0.9 μM, the latter of which is the same affinity as FMNL2_N_-Cdc42 binding. The thermodynamic parameters of the interactions are listed in Table 2. (**e**) Phase-contrast (left) and widefield fluorescence (right) images of living B16-F1 cells expressing EGFP–FMNL2 co-transfected with myc-tagged GTPases as indicated. Note that while all expressed GTPases induce flat lamellipodia, only Cdc42 (L61) and the triple mutant of Rac1 L61 (Rac1 3x (L61)) are able to drive accumulation of full-length EGFP-tagged FMNL2 at the leading edge. Scale bar, 10 μm (valid for all panels).

The interaction map of FMNL2 binding in the complex interface with the GTPase reveals only three residues difference between Cdc42 and Rac1 (Fig. 2). We first mutated A95 in Rac1 to glutamate to allow formation of the hydrogen bond network with H222 and R261 of FMNL2 (Fig. 3a, left panel). ITC experiments, however, showed no binding, suggesting that exchange of the alanine to glutamate was not sufficient to increase the affinity to detection levels (Fig. 3b). Next, the two residues E131 and K132 in the insert helix of Rac1 were exchanged to its Cdc42 counterparts K131 and N132, respectively, introducing thereby a charge reversal for the proposed interaction with E360 of FMNL2 (Fig. 3a, right panel). The affinity of this Rac1 (E131K and K132N) double mutant increased indeed to 10.6 μM as determined by ITC (Fig. 3c). Combining all three mutations, A95E, E131K and K132N, finally raised the affinity of this Rac1 triple mutant for FMNL2 binding to 0.9 μM (Fig. 3d), exhibiting now the same affinity as the cellular activator Cdc42 (Table 2). We thus generated a gain-of-function mutation of Rac1 for FMNL2 binding by exchanging only three residues in the GTPase, of which two are in the insert helix and none in the switch regions. A minor contribution to the specificity of the interaction might come as well from K94 in Cdc42, which represents an arginine in Rac1-3 and a proline in RhoA-C. This residue can form a salt bridge with D219 of FMNL2, although the electron density is not well resolved in the crystal structure.

To test in cells whether the triple mutant was capable of interacting with and activating FMNL2, we analysed the localization of an autoinhibited, enhanced green fluorescent protein (EGFP)-labelled variant of FMNL2 (ref. 28) in the presence of Cdc42, Rac1 or the Rac1 triple mutant (Fig. 3e). In the absence of a transfected Rho-GTPase, wild-type FMNL2 tagged at the N terminus with EGFP was entirely cytosolic in B16-F1 cells^28^. As shown previously, co-expression of this FMNL2 variant with constitutively active Cdc42 (Q61L mutant) directed the formin to the cell periphery (Fig. 3e and ref. 28). Such effect was not observed for constitutively active Rac1 (Rac1 L61), in spite of prominent induction of lamellipodia, whereas co-expression of Rac1 L61 triple mutant (A95E, E191K and K192N) caused prominent accumulation of EGFP–FMNL2 at the cell periphery (Fig. 3e). These data demonstrate that Rac can be functionalized to activate FMNL2 *in vivo* solely through enabling its interaction with the formin. Interestingly, this specificity is underscored by the finding that the Rac triple mutant maintained the capability of interacting with the WAVE complex, but was still unable to bind to the Cdc42-effector N-WASP (Supplementary Fig. 5).

### Myristoylation and a basic patch mediate membrane binding

**Figure 4:**
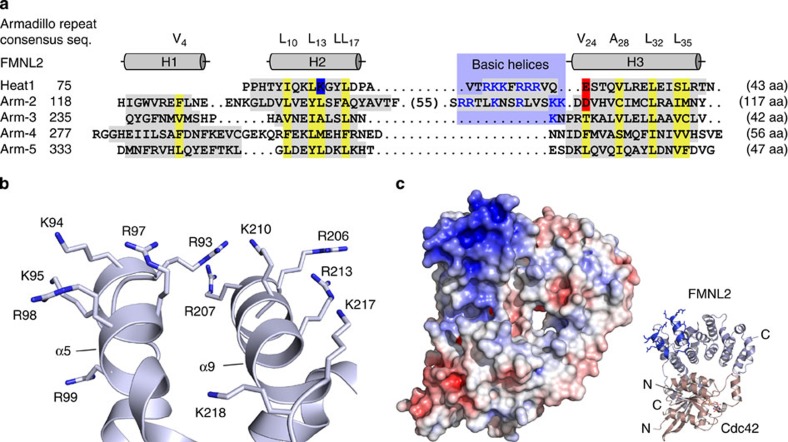
A surface patch of 12 basic residues is inserted into the FH3 domain. (**a**) Armadillo repeat structure of the FMNL2 FH3 domain. α helices are shaded grey with the armadillo repeat consensus sequence printed on top as reported^59^. Hydrophobic residues that mediate the core scaffold within the armadillo repeats are marked yellow. Positively and negatively charged residues are labelled blue and red, respectively. Two inserted sequence stretches in the first and second repeat preceding the third helix H3 constitute a basic patch. (**b**) Close-up view on the 12 arginine and lysine residues in the non-canonical helices α5 and α9 that form the basic surface patch in the FH3 domain. (**c**) Electrostatic surface potential of the Cdc42–FMNL2_N_ complex displayed from -15 *k*_B_T (red) to +15 *k*_B_T (blue). A large basic surface patch is formed by the two non-canonical helices.

The neighbouring helices α5 and α9 interrupting the canonical armadillo repeat fold contain two basic patches composed of six positively charged residues each (Fig. 4a). The sequence motifs ^93^RKKFRRR on the first and ^206^RRTLKNSRLVSKK on the second helix assemble in a way that all 12 basic moieties are exposed on the surface of FMNL2 at a position adjacent to the Cdc42 binding site (Fig. 4b). Together, these residues constitute a strongly positively charged patch as seen from the electrostatic surface representation (Fig. 4c). The basic sequence is conserved in all three mammalian FMNL homologues, with 10 and 11 positively charged residues in FMNL1 and FMNL3, respectively (Supplementary Fig. 4). The flexible loop of 55 residues that is not resolved in the electron density map of the FMNL2_N_–Cdc42 structure is directly preceding the second basic helix α9. Interestingly, this loop is missing in one splice variant of FMNL3, isoform 2, but otherwise conserved in all FMNLs (Supplementary Fig. 4).

**Figure 5:**
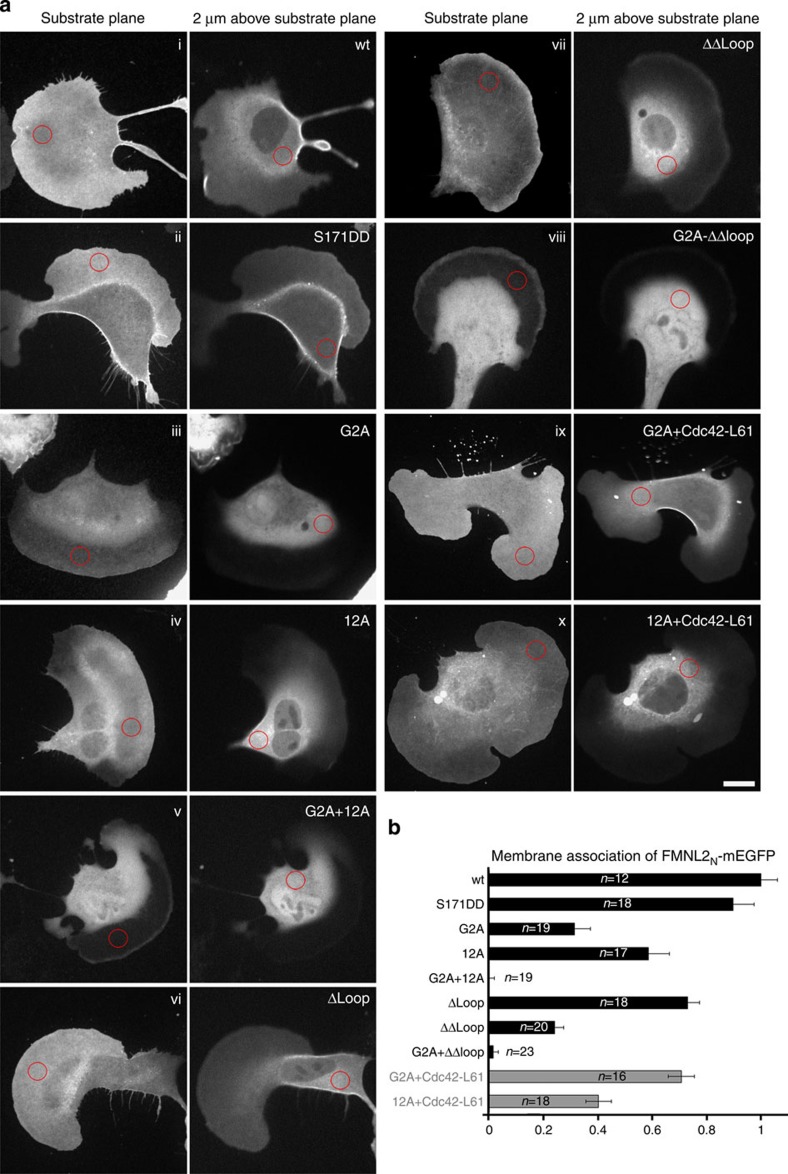
Several motifs mediate the membrane association of FMNL2. (**a**) Representative spinning disc confocal images of live B16-F1 cells expressing FMNL2_N_–EGFP constructs (1–385 aa; upper left) or mutants hereof as indicated. Each panel shows two acquisition planes of the same cell. The left shows the substratum plane, the right shows the plane 2 μm above the substrate. Circular regions indicate regions taken for intensity measurements of the plasma membrane (substrate plane) and cytosol (2 μm above substrate plane). Scale bar, 20 μm. (**b**) Quantitation of membrane association of FMNL2_N_–EGFP constructs shown in **a**. Values from intensity measurements derived from wt and G2A+12A mutant were normalized to 1 and 0, respectively, as explained in Methods. *n* denotes the number of cells quantified and data are presented as mean±s.d.

As FMNL2 is myristoylated and likely activated by membrane-associated Cdc42, we analysed the contribution of these two membrane targeting motifs for localization of the formin at the plasma membrane. Fluorescent mEGFP was fused C-terminally to FMNL2_N_ 1–385, such that the N-terminal MGxxxS motif of FMNL2 is accessible for co-translational myristoylation in cells. Expression plasmids were transfected into B16-F1 melanoma cells and only cells with a weak protein expression level were considered for evaluation. Relative membrane association of each construct was determined by two alternative methods, revealing highly similar results (Fig. 5 and Supplementary Fig. 6). Wild-type FMNL2_N_ and the phosphorylation mimic FMNL2_N_ (S171DD) localized similarly well to the plasma membrane (Fig. 5a and Supplementary Fig. 6a, i and ii). This observation is not supporting the hypothesis that phosphorylation of S171 could be a release mechanism for FMNL2 membrane dissociation, as reported, for example, for myristoylated MARCKS proteins^42^. Mutation of all 12 basic residues in the insert helices to alanine (12A) reduced membrane association significantly, but not as potently as the myristoylation-defective G2A mutant (iii and iv). Only combined mutation of both membrane targeting motifs (G2A and 12A) resulted in fully cytosolic protein localization, establishing the cooperation of both motifs in membrane targeting of FMNL2 (v). Deletion of the flexible loop together with the second basic insert helix (Δ144–218, Δloop) reduced membrane localization only weakly. Yet, the defect was increased on combination with a deletion of the first basic insert helix (Δ93–100, Δ144–218, ΔΔloop) (vi and vii). The combination of the basic insert deletions with myristoylation deficiency (G2A+ΔΔloop) decreased membrane association even further (viii). Membrane localization of FMNL2_N_ (G2A) in B16-F1 cells was significantly increased on co-transfection with constitutively active Cdc42(Q61L) (ix). In contrast, membrane association of the basic patch mutant FMNL2_N_ 12A was reduced even further on co-expression of Cdc42(Q61L) (x). These observations suggest that a precast conformation of FMNL2_N_ at the membrane mediated by the large basic surface patch supports a productive interaction with Cdc42. A quantification of the FMNL2 membrane binding features according to the evaluation scheme shown in Supplementary Fig. 7 is provided in Fig. 5b. The various protein constructs used are displayed in Supplementary Fig. 8. Co-sedimentation and PIP-strip assays of the FMNL2 N-terminal domains confirmed the membrane association *in vitro*, showing that the association is driven by myristoylation and electrostatic interaction (Supplementary Fig. 9).

### FMNL1–Cdc42 assembles into a symmetrical dimer

**Figure 6:**
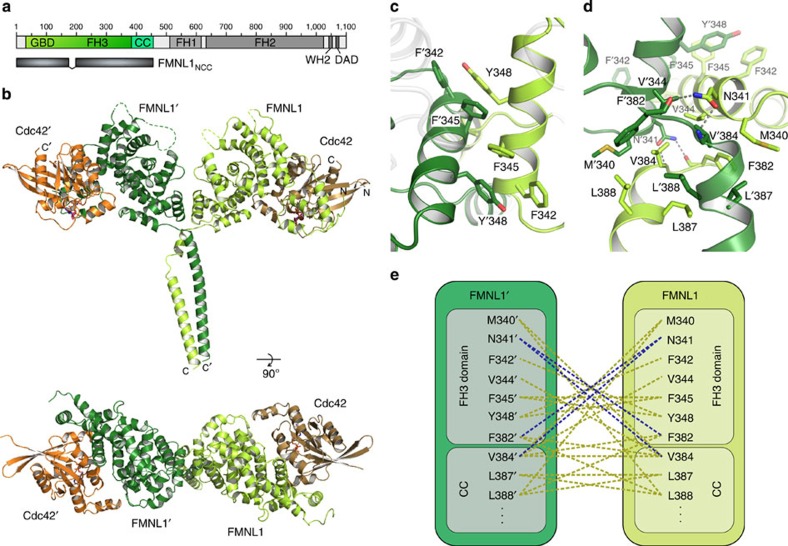
Assembly of the FMNL1_NCC_–Cdc42 complex dimer. (**a**) Domain architecture of human FMNL1. A protein construct encompassing the N-terminal domains GBD-FH3-CC including a 27-residue-encompassing deletion within the flexible loop was used for crystallization with Cdc42. (**b**) Structure of the FMNL1_NCC_–Cdc42
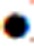
GppNHp heterodimer at 3.8 Å resolution. The protein complex adopts an umbrella-shaped structure by the C-terminal coiled coil domain. Of the coiled coil domain, five hepta-repeats and a stammer of three residues are resolved in the crystal structure. A view from the top shows the elongated shape of the dimer with the two GTPases opposing each other distantly. (**c**) Close up of the dimer interface. Two phenylalanines and a tyrosine on the first helix of the last armadillo repeat form the core assembly of the formin homodimer. (**d**) Close up of the linker element between the FH3 domain and the adjacent coiled coil domain. N341 mediates key interactions with F382 and V384 of the opposing chain. (**e**) Interaction scheme of the FMNL1 dimer interface. Hydrophobic interactions are shown in ochre, and polar interactions are shown in blue dashed lines.

Diaphanous-related formins Dia, Daam and FMNL, but not FHOD, contain dimerization elements following the regulatory GBD-FH3 domains^4^. This dimerization element comprises either a three helical bundle, designated as dimerization domain (DD) as found in mDia1 (refs 17, 18), or a coiled coil domain directly following the armadillo repeats. We succeeded in crystallizing the complex of Cdc42
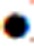
GppNHp with FMNL1_NCC_ (1–458, Δ172–198), the last canonical armadillo repeat of which ends at position 380 (Fig. 6a). The complex was refined to 3.8 Å resolution (Table 1), allowing information on the overall assembly of the four subunits and the formation of the coiled coil backbone (Fig. 6b). The interaction network including the specific interactions of the insert helix seen for the FMNL2–Cdc42 complex are similarly observed in the FMNL1–Cdc42 structure, which is reflected by an root mean square deviation value of 0.63 Å over the aligned amino acids (Supplementary Fig. 10a).

Surprisingly, the FMNL1 FH3 structure is directly continuing into a parallel coiled coil segment starting at position 384, which could be resolved up to position 422 (Fig. 6b). The mostly hydrophobic dimer interface of the FH3 domain is essentially formed by two phenylalanines and one tyrosine as seen in the electron density map (Supplementary Fig. 10b). These residues are located on the first helix α17 of the fifth armadillo repeat and supported by neighbouring residues (Fig. 6c,d). The typical knobs-into-holes packing of the coiled coil assembly starts with its first amino acid V384 and is continued in the classical abcdefg hepta-repeat scheme, with residues at position a and d forming key interactions of the coiled coil^43^. An overview of the symmetric interaction pattern of the FMNL1 homodimer is shown in Fig. 6e. Although the crystal structure of the coiled coil domain is only resolved for the N-terminal 60% of this segment, its continuation can be anticipated from a sequence analysis up to the beginning of the proline-rich FH1 domain (Supplementary Fig. 11a,b). Analysis of the hepta-repeat structure suggests that the coiled coil element is interrupted by discontinuities, known as 'stammer' and 'stutter' motifs^43^. Such variations from the canonical hepta-repeat sequence by insertions of three and four residues, respectively, are known for many coiled coil containing proteins, as for example, myosin, possibly allowing proper folding of the domain and some conformational flexibility. The coiled coil element encompasses 101 amino acids in FMNL2 and FMNL3, spanning ∼152 Å in length, as derived from a modelling approach based on coiled coil elements containing similar stutter and stammer elements. In contrast, the coiled coil domain of FMNL1 contains only an ∼70 residues and misses the third segment but contains an elongated poly-proline FH1 region instead (Supplementary Fig. 11a,b).

### The membrane targeting motifs align on a convex surface

**Figure 7:**
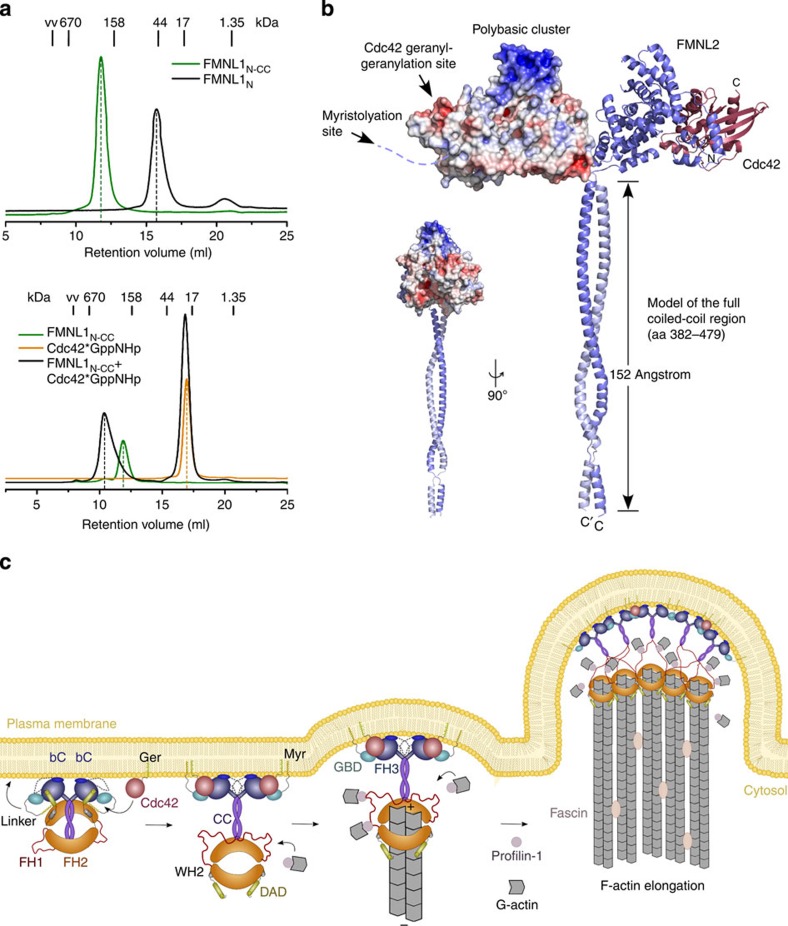
Model of FMNL2-mediated cell protrusions at the leading edge. Size exclusion chromatography of FMNL1 complexes. Expanding the domain boundaries from FMNL1_N_ (1–385, GBD–FH3) to FMNL1_NCC_ (1–458, GBD-FH3-CC) leads to dimerization of the formin (upper panel). Addition of Cdc42
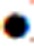
GppNHp to FMNL1_NCC_ increases the size of the heterodimer even further (lower panel). (**b**) Atomistic model of the full FMNL2 N-terminal domains (1–479) as a coiled coil dimer in complex with Cdc42. One heterodimer is shown as electrostatic surface representation while the other chain is displayed as a cartoon. In an extended conformation, the coiled coil spans 152 Å, leading to an 'umbrella'-shaped conformation with a total size of 210 Å in height, 153 Å in width and 50 Å in depth. The complex twisted by 90 °C is shown as insert. (**c**) Cartoon model of FMNL2-mediated protrusion outgrowth. FMNL2 is capable of accumulating at the tips of both filopodia and lamellipodia, distal to and coincident with polymerizing actin networks and bundles, respectively (Supplementary Fig. 13). This may be achieved through a sequence of consecutive steps: in the inactive state, FMNL2 associates to the plasma membrane by its N-terminal myristoylation (Myr) and the FH3 basic cluster (bC). On activation by geranylgeranylated (Ger) Cdc42, the autoregulatory DAD is displaced and the FH1 and FH2 domains will become accessible for actin filament elongation. The convex conformation of the FMNL2–Cdc42 complex may sustain membrane protrusion by forming a membrane dome (Supplementary Fig. 14). Multiple FMNL2 dimers may assemble to stabilize lamellipodial and filopodial membrane bulges.

The elongated conformation of the FMNL1 dimer was confirmed by size exclusion chromatography experiments showing an increase from 44 kDa in apparent mass for FMNL1_N_ 2–385 to >200 kDa for FMNL1_NCC_ 1–458 (Fig. 7a). This size was increased further on addition of triphosphate-bound Cdc42 to the complex, in agreement with binding of Cdc42 to the oppositely positioned formin GBD. This 'dimer of heterodimers' assembly leads to an umbrella-shaped structure of the FMNL1_NCC_–Cdc42 complex that is about 153 Å in width and 210 Å in height, based on modelling of the entire coiled coil segment, with an ∼50 Å in depth (Fig. 7b). A similar increase in size indicating protein dimerization was observed for the N-terminal FMNL2 constructs on inclusion of the coiled coil domain into the expression constructs (Supplementary Fig. 11c).

Binding of Cdc42 to the FMNL1 dimer leads to formation of a convex surface that contains the membrane interaction motifs (Fig. 6b). The position of the membrane interaction motifs in FMNL2 on activation by the GTPase can be localized based on the FMNL1_NCC_–Cdc42 complex structure. Strikingly, the N-terminal myristoylation site of FMNL2, the C-terminal geranylgeranyl moiety of Cdc42 and the 12 residues encompassing a polybasic patch all align on the convex surface of the heterodimer, leading in total to 6 symmetrically arranged membrane interaction motifs in the N-terminal domains of the activated formin complex (Fig. 7b). This assembly of the overall structure could support actin-based protrusions by orienting the formin appropriately towards the membrane and forming an outward membrane bulge for lamellipodia and filopodia growth (Fig. 7c).

## Discussion

In this study, we show that the binding interface of the GTPase Cdc42 to its effector proteins FMNL1 and FMNL2 covers all five armadillo repeats of the formin in addition to the N-terminal GBD. Particularly, the Rho-GTPase-specific insert helix α4 of Cdc42 is involved in interactions with the fourth and fifth armadillo repeat of FMNL2, contributing thereby to the specificity of the formin recognition. While residues in the switch I and II regions of Rho GTPases are largely conserved within this GTPase family, amino acid changes occur among the four residues in the insert helix α4 that we identified here to contribute to effector binding. This is different from observations in the Ras-family of GTPases, where single amino acid changes in the switch regions (namely the 'effector loop' switch I) account for the specificity in signal transduction pathways by their defined interactions with downstream effector proteins^44^. Based on this interaction scheme, the specificity of Rho, Rac and Cdc42 GTPases for their effector proteins and thereby for distinct actin cytoskeleton phenotypes^45^ has remained unanswered. One recent example is the binding of the inverted BAR protein IRSp53 by its CRIB domain to Cdc42, where a proline-rich sequence following the CRIB domain interacts with a surface patch adjacent to the switch regions^46^. With the structures of the Cdc42–FMNL1 and -2 complexes determined here we attribute the insert helix of Rho GTPases a determining function in the recognition of formin effectors and propose to consider the entire GBD-FH3 domain assembly of DRFs as the full binding unit for Rho GTPases.

It has previously been shown that wild-type Rac1 was unable to interact with FMNL formins^28^. By transferring three residues from Cdc42 to Rac1, we were able to generate a gain-of-function mutation in Rac1 for FMNL2 binding and activation (Fig. 3). Mutation of A95E in the Rho-characteristic bulge of helix α3 and mutation of two variable amino acids in the insert helix of Rac1 (E131K and K132N) converted this triple mutant into a *bona fide* FMNL2 activator, both *in vivo* and *in vitro*. This 'proof-of-principle' experiment establishes that residues outside the canonical switch I and II regions can determine the specificity of Rho GTPases for their effector proteins. While we follow here a structure-based approach to change the effector-binding specificity of a Rho-GTPase, a heuristic algorithm was previously proposed for switch-of-function mutants in Rho family GTPases, based on the classification of cell morphologies^47^.

A conformational change in the switch regions of the GTPase on activation from the GDP- to GTP-bound state is a prerequisite for effector binding. These changes are recognized by the N-terminal GBD subdomain of the formin, particularly through interactions of its second helix with switch II of the GTPase. The distant armadillo repeats instead form ionic interactions with polar residues at the C terminus of the Cdc42 insert helix, contributing thus to an entire network of polar interactions in the interface of the two proteins. This mode of complex formation involving all armadillo repeats in conjunction with the GBD is likely to exist in all Rho-GTPase–formin interactions. The interaction might have previously not appeared due to crystal packing arrangements. The missing contribution of the C-terminal armadillo repeats to the specificity of the GTPase–formin interaction may indeed account for ambiguities of the effector-binding specificity in previous studies that were based on shortened domain fragmentations. To our knowledge, this is the first description of an involvement of the Rho-specific insert helix in binding and recognition of a downstream effector. An overview of binding interfaces of GDIs, GEFs, GAPs and effector proteins derived from different Rho GTPases is displayed in Supplementary Fig. 12.

FMNL2 is effectively targeted to lipid membranes by two membrane interacting motifs. While myristoylation is recognized from the primary sequence as an inherent N-terminal lipidation signal that occurs on protein translation, the surface patch composed of 12 positively charged residues in the FH3 domain of FMNL2 was only identified here on its structure determination. Myristoylation is often accompanied by amphipathic helices or adjacent cysteine palmitoylation motifs that strengthen the membrane association or may allow for reversible S-acylation cycles, respectively. Such additional motifs are not present in FMNL2 or FMNL3, as neither an accumulation of basic residues nor a cysteine is contained in the N-terminal sequence. Systematic analysis of the membrane interacting features of FMNL2 revealed that membrane binding decreases most significantly in the myristoylation-defective G2A mutant, whereas deletion of the polybasic patch results in a milder membrane association defect in cells (Fig. 5). Yet, while myristoylation sticks an acyl chain into the lipid membrane, the spacious basic surface patch in the FH3 domain of FMNL2 might help to orient the formin at the membrane leaflet. A precast orientation could facilitate the interaction with the GTPase at the membrane, which could in turn explain why active Cdc42 is able to fully restore membrane association of myristoylation-deficient FMNL2, whereas the polybasic FMNL2 12A mutant is not as effectively recruited to the membrane by the GTPase. We could, however, not find any indication for a myristoylation switch mechanism in FMNL2 on interaction with Cdc42. Such mechanism would imply that the myristate becomes exposed for insertion into the membrane only when it is displaced from a binding site on the formin, for example, on activation by the GTPase. This notion is consistent with the observation that FMNL2 is also capable of targeting the cell periphery in Cdc42-deficient cells^28^.

Among the four mammalian DRF families Dia, Daam, FMNL and FHOD, FMNL2 and FMNL3 are the only two orthologues that contain lipidation motifs, indicating their fate for membrane association. DRFs are activated by Rho GTPases that are C-terminally prenylated by either farnesylation or geranylgeranylation. The GTPases in turn become activated by guanine nucleotide exchange factors at specific membrane compartments, thus directing the formin to subcellular locations on activation. On binding and activation of FMNL2, Cdc42 contributes a potent third membrane targeting motif to the active formin–GTPase complex. As FMNL2 contains itself two membrane association motifs it is tempting to speculate that robust and stable membrane association is intimately linked to its function in lamellipodia and filopodia formation (Supplementary Fig. 13). The proposed forces exerted on formins while elongating actin filaments^48^ may indeed require a tight membrane association, mediated for FMNL2–Cdc42 by three targeting motifs that are spread over the surface of the protein complex. A model of active, membrane-bound FMNL2 on actin elongation is shown in Supplementary Fig. 14.

FMNL1 assembles into a symmetrical homodimer by its coiled coil domain following directly the armadillo repeats of the FH3 domain. The parallel long coiled coil region forms a second dimerization unit aside from the C-terminal head-to-tail dimer of the actin-polymerizing FH2 domain^49^. The long coiled coil element encompasses ∼70 residues in FMNL1 and about 100 residues in FMNL2 and FMNL3. It is interrupted by stammer and stutter elements of three and four residues, respectively, as known, for example, from myosins. These discontinuities might help folding the repetitive hepta-repeat chain into the precise periodic structure and add some flexibility to the rigid chains. The dimer interface is complemented by a homomeric assembly of the last armadillo repeat, suggesting a fixed arrangement of the two opposing FH3 domains. The Cdc42-bound FMNL complex thus assembles into an umbrella-shaped structure with a convex protein surface that harbours the three membrane binding sites of each heterodimer and an extended coiled coil element that might function as a place holder.

From the spacious conformation of the dimeric, N-terminal FMNL1_NCC_ domain assembly (Fig. 7), it is difficult to envision how in the autoinhibited state the very C-terminal DAD interacts with the FH3 domain, considering the extended coiled coil element in between these two domains. Full-length, recombinant FMNL1 protein expressed from insect cells behaves as a dimer in size exclusion chromatography experiments (data not shown). At the plasma membrane, however, the FH2 domains, for example, of FMNL2, could dimerize with other FMNL2 dimers, thereby forming homogeneous, large clusters of this protein. Interestingly, supramolecular polymer structures have previously been described for other actin assembly machines, dependent on multivalent protein interactions, such as in the phospho-nephrin–Nck–N-WASP signalling module operating in Arp2/3 complex activation^50^. Multimer assemblies of FMNL2 dimers that associate by the coiled coil domain in *cis*- and by the FH2 domain in *trans*-configuration might generate the critical concentration of formin proteins required for effectively promoting filopodial and lamellipodial protrusions. Future studies will be designed to probe the effect of FMNL2 clustering at the membrane and curvature shaping for membrane outgrowth.

## Methods

### Plasmid cloning, protein expression and purification

The coding sequences of the N-terminal regions of human FMNL1 (isoform 1) and human FMNL2 (isoform 2), UniProt accession numbers O95466 and Q96PY5, were amplified from the full-length genes with primers containing NcoI and EcoRI restriction sites at the 5′ and 3′ ends, respectively. For FMNL1 protein expression constructs, a synthetic complementary DNA codon optimized for expression in insect cells (GeneArt, Regensburg) was used. FMNL1, FMNL2 and Rho-GTPase constructs were cloned in a pGEX-4T1 prokaryotic expression vector (GE Healthcare) modified with a tobacco etch virus protease cleavage site. Site-directed mutagenesis of FMNL2 and several Rac1 variants was performed using the mega primer method. All plasmids were confirmed by DNA sequencing prior to protein production.

Proteins were expressed in bacterial strains BL21-CodonPlus(DE3)-RIL, Rosetta(DE3) or B834 and purified as described, with certain modifications^28^. Briefly, plasmids encoding the desired N-terminal glutathione *S*-transferase (GST)-fusion constructs were transformed in Rosetta (DE3) cells and expressed at 293 K for 16 h using the overnight auto-induction expression method. Cells were harvested in lysis buffer (20 mM Tris/HCl pH 8.0, 500 mM NaCl, 10% glycerol, 1 mM dithioerythritol (DTE)) and lysed using a microfluidizer. Fusion proteins were isolated with GSH Sepharose FastFlow (GE Healthcare) affinity chromatography methods. The GST-tag was cleaved off with tobacco etch virus protease and separated from the desired proteins by gel filtration runs. FMNL1 proteins 1–385, 1–458, 1–458 (Δ172–198), and FMNL2 proteins 1–379, 1–379 (S171DD) and 1–489 were aliquoted in storage buffer, snap frozen in liquid nitrogen and stored at 193 K. Constructs corresponding to FMNL2 1–379 and FMNL1 1–458 were termed FMNL2_N_ and FMNL1_NCC_, respectively. Protein homogeneities and identities were confirmed by analytical gel filtration analysis, SDS–PAGE experiments and electrospray ionization mass spectrometry. Selenomethionine-labelled proteins were expressed in B834 cells using the methionine biosynthesis inhibition method and purified as described above in buffers containing 5 mM DTE.

Rho GTPases were expressed in *Escherichia coli* cells as GST-fusion proteins without the C-terminal hypervariable region, and purified as described followed by nucleotide exchange for the non-hydrolysable GTP analogue GppNHp^51^. Reverse-phase C18 HPLC column was used for monitoring nucleotide loading status of the GTPases.

### Complex formation and crystallization

The low affinity of interactions of active Cdc42 with N-terminal FMNL1 or FMNL2 fragments did not allow complex formation following co-purification strategies. Therefore, complex formations were carried out by equimolar mixing of N-terminal FMNL1 (1–458, Δ172–198) or FMNL2 (1–379) fragments with GppNHp-loaded Cdc42 (1–179) in high salt buffer (20 mM Tris/HCl pH 8.0, 500 mM NaCl, 5 mM MgCl_2_ and 1 mM DTE) followed by an overnight dialysis at 277 K in buffer C (20 mM Tris/HCl pH 8.0, 150 mM NaCl, 5 mM MgCl_2_ and 1 mM DTE). For crystallization experiments, the protein solutions were concentrated to 15 mg ml^−1^ (FMNL2_N_/Cdc42
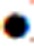
GppNHp) and 30 mg ml^−1^ (FMNL1_NCC_(Δ172–198)–Cdc42
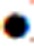
GppNHp).

Crystals of FMNL2_N_–Cdc42
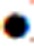
GppNHp were obtained in conditions consisting of 20 mM Tris/HCl (pH 8.0), 14% (v/v) PEG 3350 and 0.25 M magnesium acetate (FMNL2_N_–Cdc42) using the hanging drop vapour diffusion method at 293 K. The FMNL1_NCC_(Δ172–198)–Cdc42
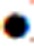
GppNHp complex crystallized in 17% (v/v) PEG 3350 and 0.16 M tri-ammonium citrate (FMNL1_NCC_–Cdc42). Crystals of SeMet-substituted FMNL2_N_ in complex with native Cdc42 were obtained essentially as described for the native protein complex, but in reservoir solution with 5 mM DTE. For cryo-protection, single crystals grown to a size of 160 × 150 × 100 μm (FMNL2_N_–Cdc42) and 80 × 80 × 20 μM (FMNL1_NCC_–Cdc42) were transferred into a solution containing the mother liquor components and additional 20% ethylene glycol as cryo-protectant. After an incubation period of 30 s, crystals were flash cooled in liquid nitrogen.

### Data collection and processing

Native diffraction data of the FMNL2_N_–Cdc42
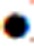
GppNHp complex were collected at 2.5 Å at cryogenic temperature (100 K) and data sets of the selenomethionin-labelled complex were collected at 3.5 Å. Crystals of the dimeric FMNL1_NCC_(Δ172-198)–Cdc42
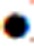
GppNHp complex diffracted up to 3.8 Å. All data sets were recorded with a MAR 225 CCD detector at X06SA beamline of the Swiss Light Source (SLS, Paul Scherrer Institute, Villigen, Switzerland) and processed with the XDS package^52^. Data collection and refinement statistics are summarized in Table 1.

### Structure determination and refinement

The structure of FMNL2_N_–Cdc42
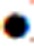
GppNHp was solved by phase combination of a native data set and single-wavelength anomalous diffraction data collected at the selenium absorption edge. For the native complex structure, maximum likelihood molecular replacement was performed with Phaser from the CCP4 package^53^ using Cdc42 of the mDia1_TSH_–Cdc42
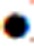
GppNHp structure (PDB 3EG5, Chain A) as search model. Nine out of 12 selenium sites in FMNL2_N_ were found in the data set of SeMet-labelled FMNL2_N_–Cdc42. Phase combination was carried out using Phaser-EP/Phenix^54^. The output file was merged with the native and SeMet-labelled data set and the initial phases were extended to the higher-resolution native data set in DM (CCP4; ref. 55). The structural model of FMNL2_N_–Cdc42 was manually built into the resulting electron density map using COOT^56^, and ligands (GppNHp, Mg^2+^) were also added manually. The resulting model was refined in REFMAC5 of the CCP4 programme suite^57^ and in Phenix.refine^54^. The solved FMNL2_N_–Cdc42 structure was taken as a search model for molecular replacement in Phaser (CCP4; ref. 53) to obtain an initial model of the dimeric FMNL1_NCC_(Δ172–198)–Cdc42
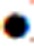
GppNHp complex structure. This structure was refined using REFMAC5, Phenix.refine with the TLS, as well as NCS option and COOT. Ramachandran statistics for FMNL2_N_–Cdc42
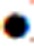
GppNHp and FMNL1_NCC_–Cdc42
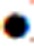
GppNHp confirmed geometries of all residues in the favoured region, with no outliers. Protein interfaces were calculated in PDBePISA^58^. All protein representations were created using the PyMOL software (DeLano Scientific LLC).

### Isothermal titration calorimetry

Interactions of FMNL proteins with the GTPases Cdc42 (aa 1–179), Rac1 (aa 1–177) and Rac1 mutants or RhoA (aa 1–193) were performed by ITC using a MicroCal iTC200 microcalorimeter (GE Healthcare) as described^59^. Measurements were carried out in 20 mM Tris/HCl buffer (pH 8), 150 mM NaCl, 5 mM MgCl_2_, 1 mM DTE at 298 K. GTPases at a concentration of 500 μM were stepwise injected from the syringe to 50 μM FMNL placed in the measurement cell. The change in heating power was observed over the reaction time until equilibrium was reached. Data were analysed using the software provided by the manufacturer.

### Size exclusion chromatography

Analytical gel filtrations of FMNL1, FMNL2 and Cdc42 proteins were performed using a multicomponent Waters 626 LC system (Waters, MA) equipped with a Superdex S200 (10/300 GL) column (GE Healthcare). Typically, 100 μg of each protein were loaded onto the column that was equilibrated in 20 mM Tris/HCl (pH 8.0), 150 mM NaCl, 1 mM DTE buffer (supplemented with 2.5 mM MgCl_2_ in the presence of Rho GTPases) prior to injection of the protein samples. Gel filtrations were run at a flow rate of 0.5 ml min^−1^ at 293 K. The optical density was monitored at a wavelength of 280 nm over the time course of the experiment. Gel filtration experiments were performed repeatedly.

### *In vitro* GST pull-down experiments

For pull-down assays, glutathione–sepharose beads (GE Healthcare) were loaded with the respective GST-tagged Rho-GTPase and washed two times in binding buffer (20 mM Tris/HCl pH 8.0, 150 mM NaCl, 10% glycerol, 0.005% Triton X-100 and 1 mM β-mercaptoethanol). Afterwards, the GTPase-bound beads were incubated with equal amounts of FMNL1_NCC_ in 275 μl binding buffer for 30 min at 277 K and washed three times with 250 μl binding buffer. The beads were resuspended in 20 μl SDS loading buffer and interactions were analysed by SDS–PAGE analysis (8 μl of each sample loaded on an 18% SDS–PAGE gel). Pull-down experiments shown in Supplementary Fig. 5 using a rabbit Sra-1 antibody (Sra-1B) were carried out as described^60^. The rabbit N-WASP antibody (N-WASP-crib1 8593) was raised against a synthetic peptide comprising aa 193–212.

### Mammalian expression constructs and cell transfections

Full-length EGFP–FMNL2 and FMNL2–EGFP capable of N-terminal myristoylation were described in ref. 28. Fusions of EGFP with N-terminal fragments of FMNL2 (aa 1–385 and mutants thereof) were generated using restriction sites EcoRI and KpnI in pEGFP–N1 expression vector (Clontech Laboratories, Mountain View, USA) harbouring a monomeric variant of EGFP, allowing for eukaryotic expression of myristoylated, FMNL2_N_–mEGFP fusion proteins. FMNL2_N_ mutations G2A, S171DD, the 12-mer mutation R93, K94, K95, R97, R98, R99, R206, R207, K210, R213, K217 and K218 all to alanine, the loop deletion mutations Δ144–218 and Δ93–100 and combinations thereof were cloned with the mega primer method. Myc-Rac1 (L61, E95, K131 and N132) was subcloned from pGEX-2T using BamHI and EcoRI and ligated into pRK5myc. All coding plasmids were confirmed by DNA sequencing prior to transfection.

B16-F1 mouse melanoma cells were grown and transfected with Superfect (Qiagen) essentially as described^61^,^28^ or transfected with PeqFect (Peqlab, Germany) according to the manufacturer's instructions using 0.5 μg DNA and 1 μl PeqFect. B16-F1 cells were transiently transfected with expression constructs overnight and seeded on glass coverslips or WillCo wells (Amsterdam, The Netherlands) coated with laminin (Roche; 25 μg ml^−1^). Widefield microscopy was performed on cells on coverslips in an open heating chamber (Warner Instruments, Hamden, CT, USA) with heater controller (Model TC-324 B, SN 1176) at 37 °C. Cells were maintained in microscopy medium (HAM's F12 HEPES-buffered medium, Sigma-Aldrich Co.) including complete supplements.

### Fluorescence microscopy

Images from live cells shown in Fig. 5 were recorded using a spinning disc confocal microscope. Images were taken using a Nikon Eclipse Ti microscope equipped with a spinning disc confocal device (UltraVIEW VoX; Perkin Elmer), solid state 488 nm laser (Modular Laser System 2.0, Perkin Elmer), a Hamamatsu EM-CCD camera (C9100-50, Hamamatsu) and a Nikon 60 × /1.4 numerical aperture Apo objective steered by Volocity software. Cells were maintained in a climate chamber using microscopy medium.

Live-cell imaging shown in Supplementary Fig. 6 was performed with phase-contrast and widefield fluorescence optics as described^28^, using a 63 × /1.4 numerical aperture Plan Neofluar objective. For comparing localization of full-length FMNL2 capable of N-terminal myristolation with actin networks and bundles, B16-F1 cells transiently expressing FMNL2–EGFP and plated on laminin were fixed with 4% paraformaldehyde in phosphate-buffered saline (20 min) followed by brief extraction (1 min) with 0.1% Triton X-100 and counterstaining with Alexa594-conjugated phalloidin (Life Technologies). Samples were subjected to fluorescence microscopy as described^28^, and green and red fluorescence channels recorded separately and merged.

Two alternative and complementary methods were developed to quantify relative plasma membrane associations of FMNL2 N-terminal constructs, one based on confocal and the second based on widefield imaging. In case of confocal imaging shown in Fig. 5, the procedure was as follows. To distinguish between plasma membrane-associated and cytosolic protein, two different planes of each cell were recorded, one focusing on the substratum and the second 2 μm above this plane (see Supplementary Fig. 7a). Fluorescence in flat cellular regions of substrate plane images mostly reflects signals from plasma membrane-associated EGFP-tagged constructs, whereas fluorescence from a region 2 μm above the substrate plane between nucleus and plasma membrane was defined to correspond to the cytosolic region. Corresponding circular regions that were measured in each part of confocal plane using Volocity software are indicated in Fig. 5. Relative intensities were expressed as ratios of ‘plasma membrane region' and ‘cytosolic region'. Twelve or more cells were measured for each construct and averaged. For the wild-type N terminus of FMNL2, which is membrane associated, this ratio was 1.53 and normalized to 1.0. The lowest measured ratio (0.21) was obtained for mutant G2A 12A protein and set to 0 in the bar chart in Fig. 5b, to ease readability of the figure and comparison of values derived from various constructs.

Quantitation of the widefield images shown in Supplementary Fig. 6 was based on the following considerations. Fluorescence intensity of cytosolic proteins when imaged by widefield optics largely depends on cell thickness, whereas this does not hold true for factors exclusively associated with the plasma membrane. More specifically, plasma membrane-associated signals generate cell images with homogenously distributed fluorescence intensity (for example, see signal distribution of plasma membrane-associated FMNL2_N_ in Supplementary Fig. 6a, i). Images were recorded and processed using Metamorph software (Molecular Devices). To quantify the data, we divided average fluorescence intensity in the lamella, which constitutes the very flat region at the cell periphery behind ruffling lamellipodia by average intensity of the entire cell (see also Supplementary Fig. 7b). Highest and lowest ratios were normalized to 1.0 and 0, respectively.

## 

## Author contributions

S.K. expressed, purified and crystallized the proteins, collected the data and determined the crystal structures. F.K., J.B. and L.S. performed the cell biology experiments under the supervision of A.S. and K.R. For Rac1 mutant studies, S.K. and C.E. purified the proteins and performed *in vitro* binding experiments, and S.K. performed and analysed additional biochemical experiments. M.G. designed the study and wrote the manuscript together with S.K., A.S. and K.R. All authors discussed the results and commented on the manuscript.

## Additional information

**Accession codes:** Atomic coordinates and structure factor files of the FMNL2_N_–Cdc42
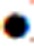
GppNHp and FMNL1_NCC_–Cdc42
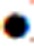
GppNHp complex structures have been deposited in the Protein Data Bank under accession codes 4YC7 and 4YDH, respectively.

**How to cite this article:** Kühn, S. *et al.* The structure of FMNL2–Cdc42 yields insights into the mechanism of lamellipodia and filopodia formation. *Nat. Commun.* 6:7088 doi: 10.1038/ncomms8088 (2015).

## Supplementary Material

Supplementary Information Supplementary Figures 1-14 and Supplementary References

## Figures and Tables

**Table 1:** Optimization of the reaction conditions for 4aa*.

**Table 2:** Thermodynamic parameters of Rho GTPase–FMNL interactions determined by isothermal titration calorimetry*.

**Titration scheme**†	***K***_**d**_ **(μM)**	**Δ*****H*** **(kcal mol**^**−1**^)	***T*****Δ*****S*** **(kcal mol**^**−1**^)	**Molar ratio n**
Cdc42 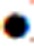 GDP to FMNL2_N_‡	—	—	—	—
Cdc42 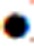 GppNHp to FMNL2_N_‡	1.89±0.23	−5.34±0.19	2.21	0.52
Cdc42 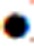 GppNHp to myrFMNL2_N_‡	5.23±1.67	−6.98±4.77	0.23	0.14
Cdc42 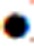 GppNHp to myrFMNL2_N_ at liposomes	1.79±0.76	−2.77±0.29	5.07	0.93
Cdc42 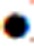 GppNHp to FMNL2_N_(Δloop)	1.34±0.17	−2.39±0.04	5.64	1.00
Cdc42 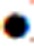 GppNHp to FMNL2_N_(S171DD)	0.79±0.17	−4.06±0.09	4.23	1.05
Rac1 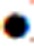 GppNHp to FMNL2_N_(S171DD)	—	—	—	—
RhoA 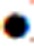 GppNHp to FMNL2_N_(S171DD)	—	—	—	—
Rac1 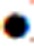 GDP to FMNL2_N_(S171DD)	—	—	—	—
RhoA 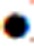 GDP to FMNL2_N_(S171DD)	—	—	—	—
Cdc42 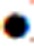 GppNHp to FMNL1_NCC_	2.55±0.49	−5.48±0.20	2.16	0.37
Cdc42 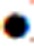 GppNHp to FMNL1_N_	6.13±1.24	−5.08±0.41	2.04	0.28
Rac1 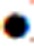 GppNHp to FMNL1_NCC_	—	—	—	—
Rac1(A95E) 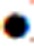 GppNHp to FMNL2_N_(S171DD)	—	—	—	—
Rac1(E131K,K132N) 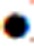 GppNHp to FMNL2_N_(S171DD)	10.65±2.76	−0.63±0.04	6.14	1.07
Rac1(A95E,E131K,K132N) 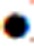 GppNHp to FMNL2_N_(S171DD)	0.90±0.47	−2.35±0.14	5.93	1.01

^*^All measurements were performed at 25 °C.

^†^FMNL2_N_ and myrFMNL2_N_ encompassed residues 2–379, FMNL2_N_(S171DD) and FMNL2_N_(Δloop) residues 1–379 (Δaa 144–218), FMNL1_N_ residues 2–385, FMNL1_NCC_ residues 2–458, Cdc42 residues 1–179, Rac1, as well as its mutants residues 1–177 and RhoA residues 1–190.

^‡^As described in ref. 28 and depicted for comparison.
