# Supplementary material for: The structure of FMNL2–Cdc42 yields insights into the mechanism of lamellipodia and filopodia formation
Source: Nat Commun. 2015 May 12;6:7088. doi: 10.1038/ncomms8088 (PMC4432619; doi:10.1038/ncomms8088)
Supplement: Supplementary Information — Supplementary Figures 1-14 and Supplementary References [file ncomms8088-s1.pdf]

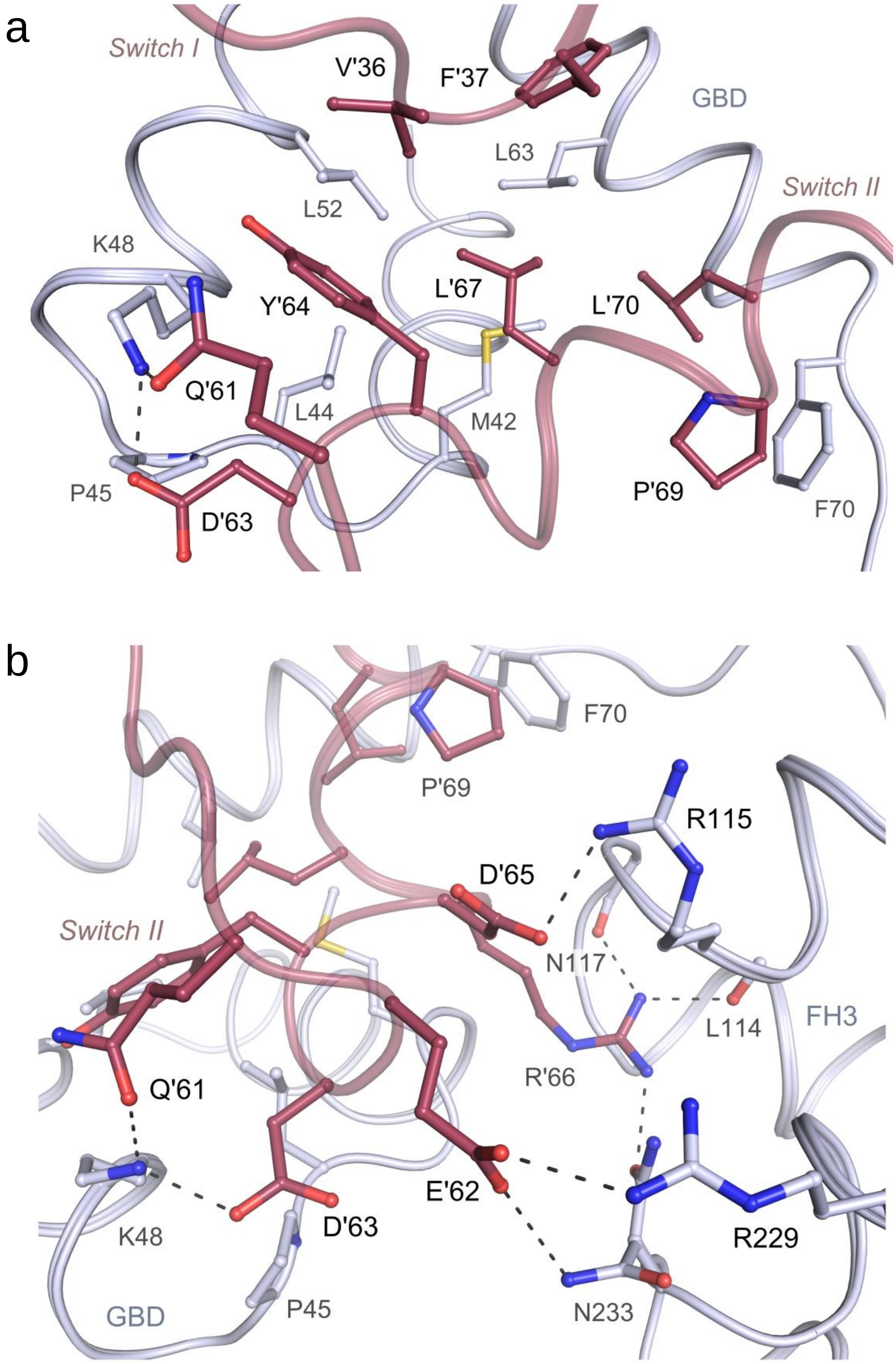

Supplementary Fig. 1: | Close-up of the interactions between Cdc42 and FMNL2.
(**a**) Interactions of switch I and switch II of Cdc42 with FMNL2. Only V'36 and F'37 of switch I in Cdc42 participate in hydrophobic interactions with the GBD of FMNL2. (**b**) Hydrogen bond network between residues Q'61, E'62, D'63, D'65 and R'66 of Cdc42 with residues of FMNL2. Q'61 and D'63 are interacting with the amino group of K48 of the GBD, E'62 interacts with R229 and N233 of the second armadillo repeat of the FH3 domain, D'65 forms a salt bridge with R115 of FMNL2 and R'66 coordinates the carboxyl groups of L114 and N117 of the first heat repeat of the FH3 domain.

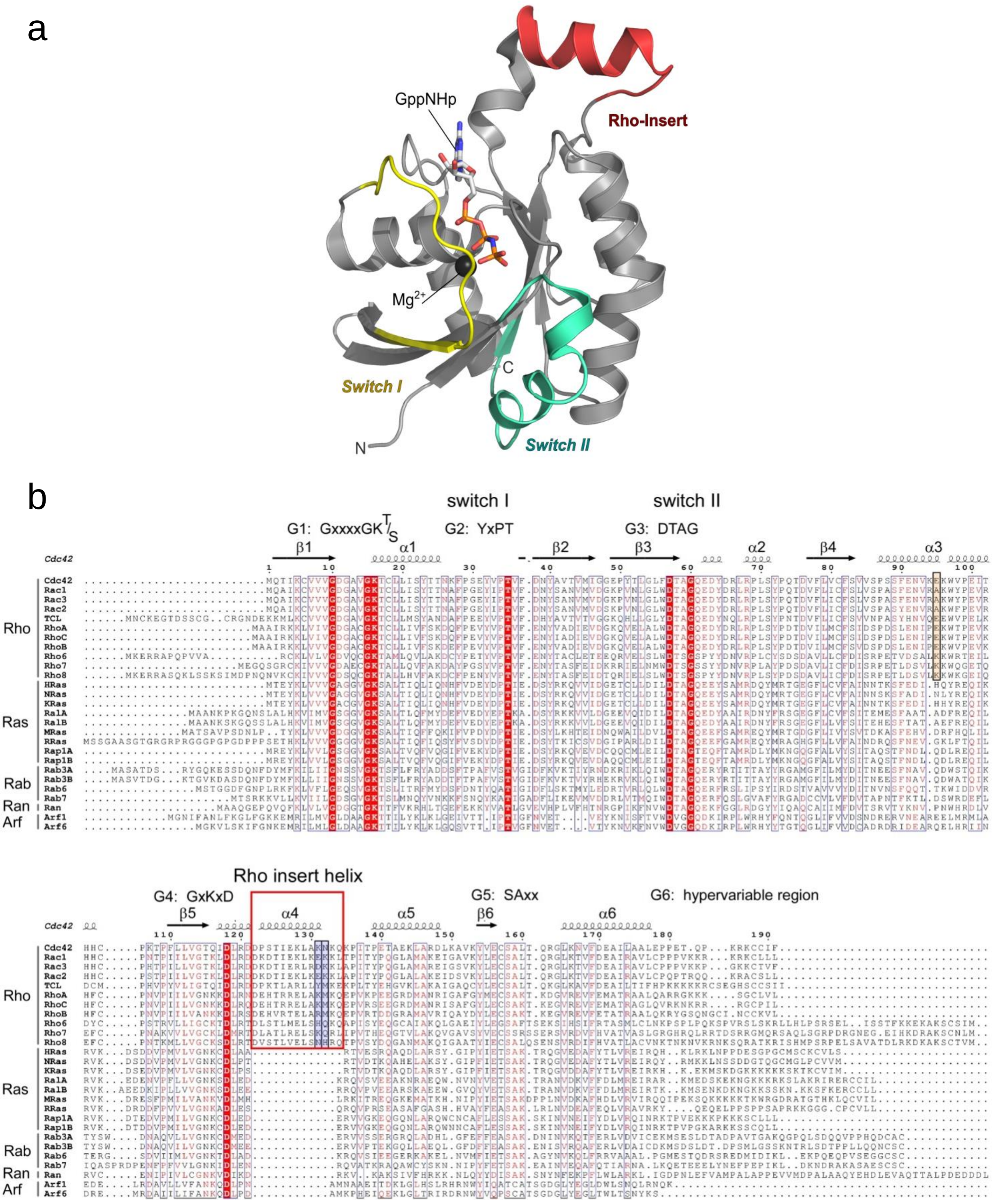

Supplementary Fig. 2 | The insert helix is a specific element of Rho GTPases.
(**a**) Structure of a triphosphate bound Rho GTPase displaying the exposed, Rho GTPase specific insert helix. (**b**) Sequence alignment of selected human Rho, Ras, Rab, Ran, and Arf GTPase families. The insert helix α4 is a specific feature of Rho GTPases. The secondary structure of Cdc42 as determine here is displayed above the sequence. The canonical G-domain elements G1 to G5 and the hypervariable region at the C-terminus mediating membrane binding are indicated[1].

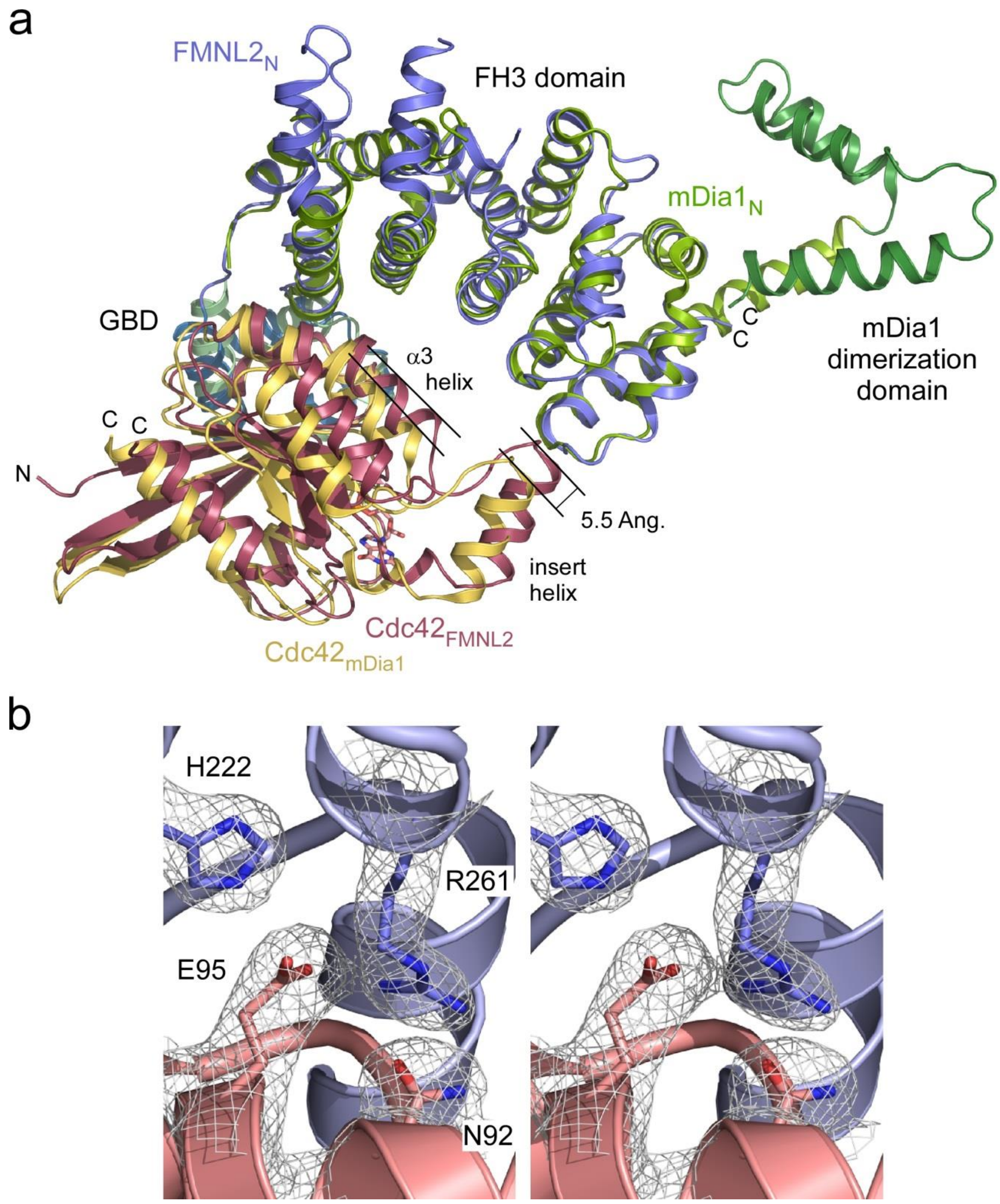

Supplementary Fig. 3 | Overlay of formin/GTPase complex structures.
(**a**) Overlay of complex structures $FMNL2_N$/Cdc42 (4YC7, this study) and $mDia1_N$/Cdc42 (3EG5, ref. 2) based on a superimposition of the FH3 domain of each formin subunit. In the FMNL2 complex the insert helix of Cdc42 comes 5.5 Å closer to the FH3 domain compared to the mDia1 complex. (**b**) Stereo image of the salt bridge formation between E95 of Cdc42 and H222 and R261 of FMNL2. The final $2F_o - F_c$ electron density map is displayed at 1σ.

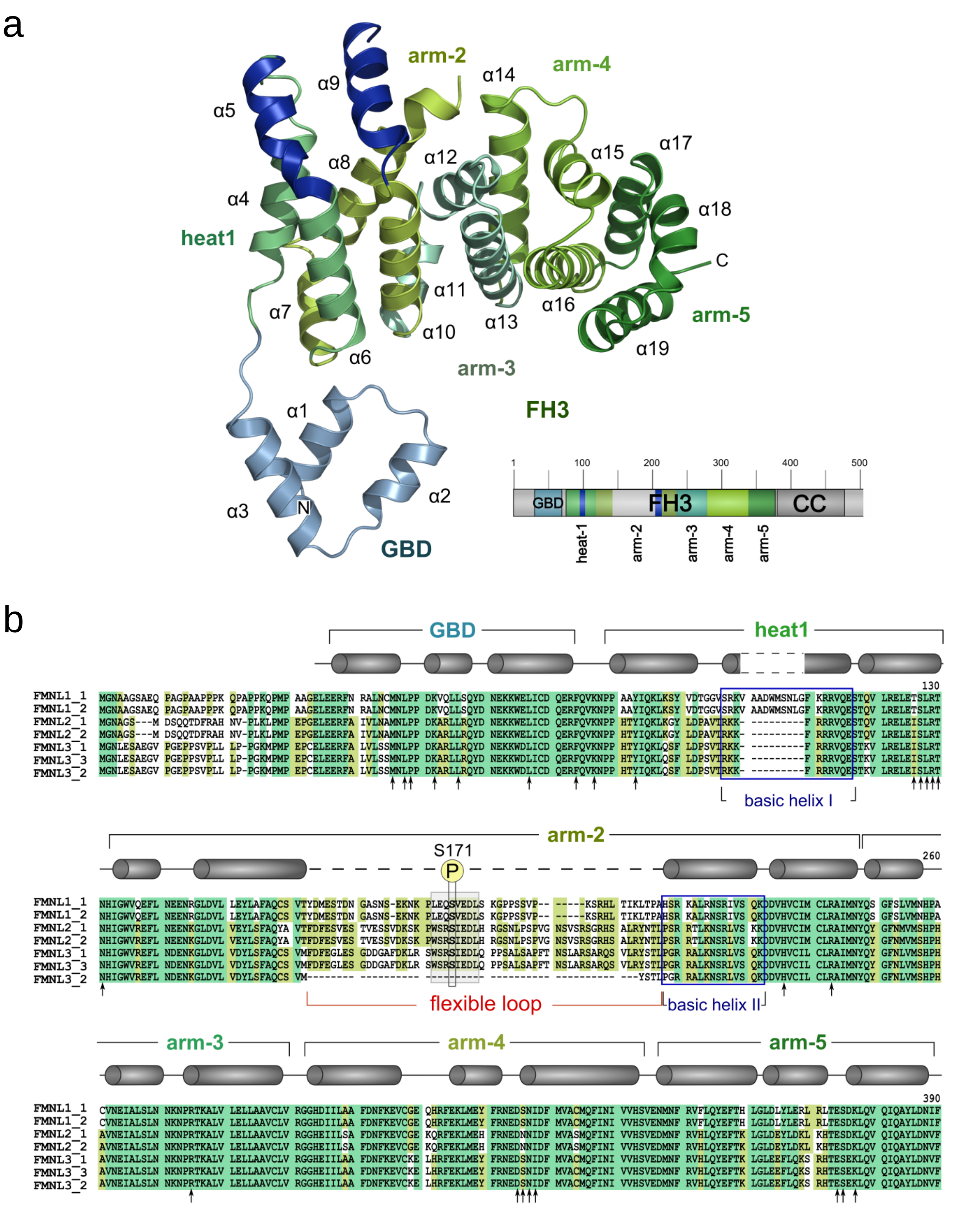

Supplementary Fig. 4 | Structure and sequence alignment of human FMNL isoforms. (**a**) Structure of the FMNL2 GBD-FH3 domain assembly derived from the Cdc42 complex. The first repeat of the FH3 domain is a heat repeat consisting of two helices only, while the following four repeats comply with the armadillo repeat fold. The two non-canonical helices ($\alpha 5$ and $\alpha 9$) containing together 12 basic residues are shown in blue. (**b**) Sequence alignment of human FMNL isoforms with the secondary structure of $FMNL2_N$ shown on top. Residues involved in the interaction with Cdc42 are marked by arrows. Displayed are UniProt sequences FMNL1-1 (O95466-1), FMNL1-2 (O95466-2), FMNL2-1 (Q96PY5-1), FMNL2-2 (Q96PY5-3), FMNL3-1 (Q8IVF7-1), FMNL3-2 (Q8IVF7-2), and FMNL3-3 (Q8IVF7-3).

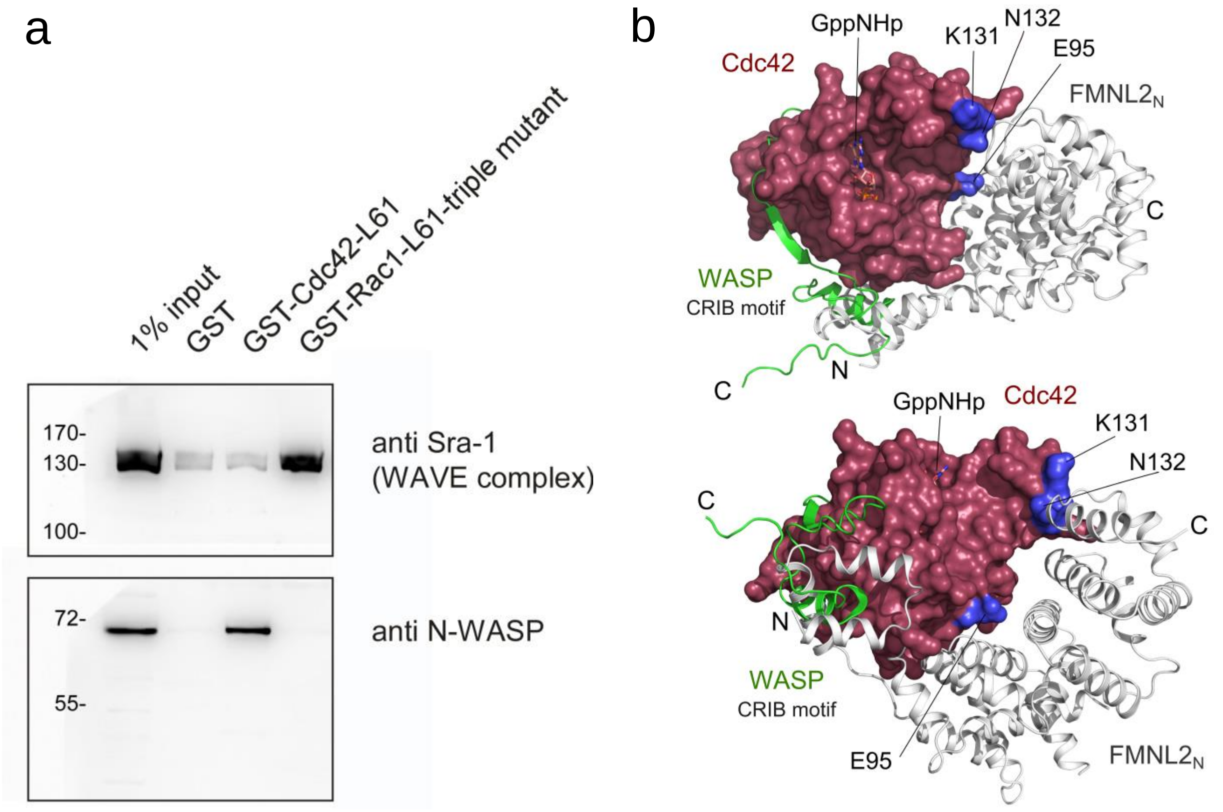

Supplementary Fig. 5 | Interaction of the Rac1 triple mutant with WAVE.

(**a**) Pull downs using GST-fusion proteins as indicated show that the Rac1-L61 triple mutant but not Cdc42-L61 retains its ability to interact with the WAVE complex, as exemplified by anti-Sra-1 Western Blotting. In contrast, and opposite to Cdc42-L61, the triple mutant lacks interaction with the bona fide Cdc42 effector N-WASP, as expected. This confirms that the gain of effector interaction of the Rac1-L61 triple mutant is restricted to FMNL proteins. GST alone was used as a control. (**b**) Superimposition of the Cdc42–WASP complex structure (1CEE, ref. 3) with the Cdc42–FMNL2 structure determined here. The three key determining residues E95, K131, and N132 (colored blue) that led to a gain-of-function mutation for FMNL2 binding when introduced in Rac1 are not involved in the binding interface of Cdc42 with WASP. In agreement with this, the triple mutation does not transform Rac1 into a WASP binding GTPase as see in the pull down assay (panel A, right lane).

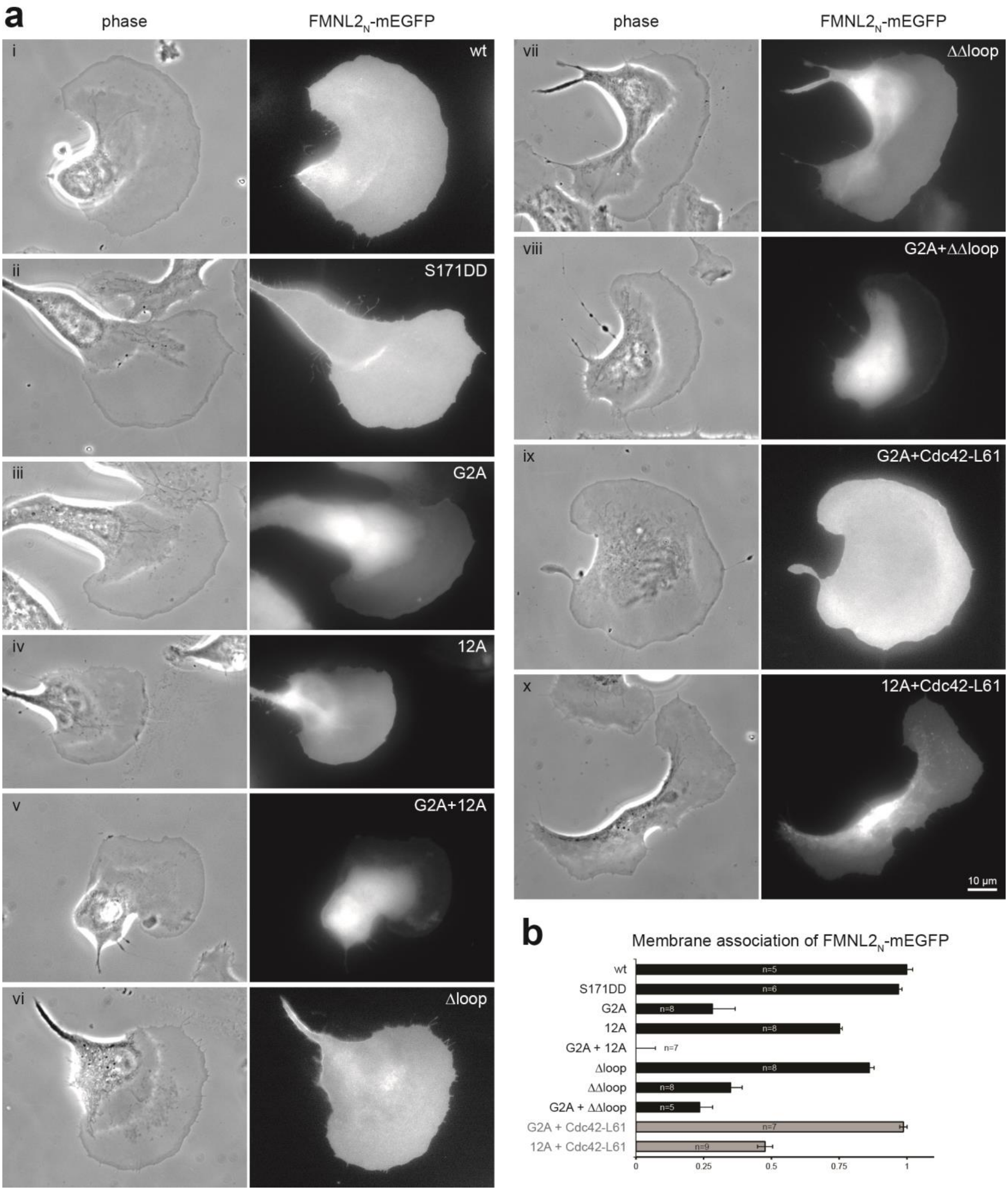

Supplementary Fig. 6 | Fluorescence microscopy of FMNL2 membrane binding.
(**a**) Representative phase contrast (left) and wide field fluorescence (right) images taken from live cell videos of B16-F1 cells expressing FMNL2$_N$-EGFP (1-385 aa; upper left panel) or mutants of the same construct as indicated. Scale bar shown is valid for all panels. (**b**) Quantitation of membrane association of FMNL2N-EGFP constructs shown in (a). Values from fluorescence measurements obtained for the wt construct and G2A+12A were set to 1 and 0, respectively, as detailed in Methods and illustrated in Supplementary Fig. 7b.

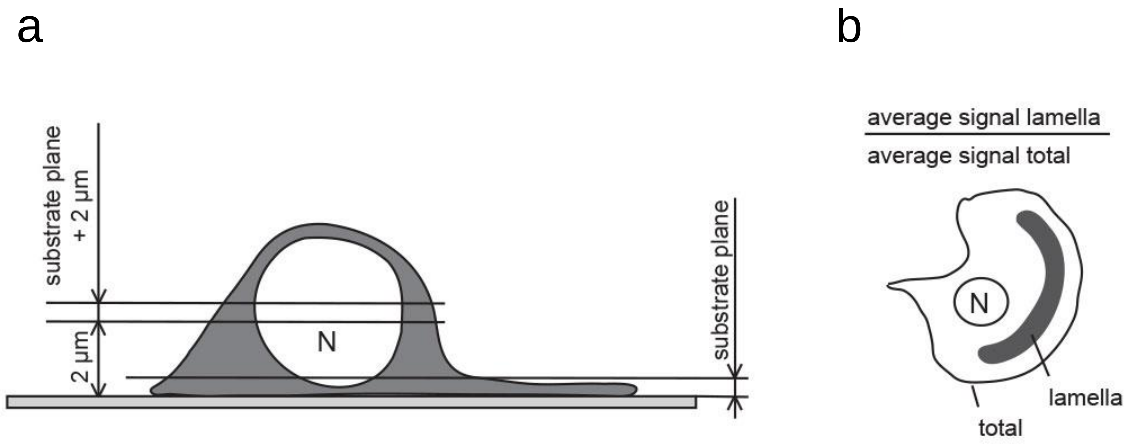

Supplementary Fig. 7 | Schematic display of fluorescence data evaluation.
(**a**) Schematic representation of quantification of FMNL2 fragment association to the plasma membrane based on confocal microscopy (related to Fig. 5). Horizontal pairs of lines depict positions of focal planes recorded for each cell analysed. Note that due to the flatness of the cell periphery (100-200 nm), fluorescence from regions measured in this area derives to a large extent from plasma membrane-associated (ventral and dorsal) molecules, whereas fluorescence from regions between plasma membrane and nucleus in the focal plane 2 µm above the substrate plane will exclusively be of cytosolic origin. As detailed in Methods, fluorescence from circular regions in each plane was measured using Volocity software. (**b**) Schematic representation of quantification of FMNL2 fragment association to the plasma membrane based on widefield fluorescence imaging (related to Supplementary Fig. 7). The area corresponding to the lamella and the total cell area were digitally drawn as depicted in the scheme and with the help of phase contrast images of live cells acquired in parallel to widefield fluorescence images (see also Methods). These regions were subsequently copied onto corresponding fluorescent images, and average fluorescence intensities computed using Metamorph software.

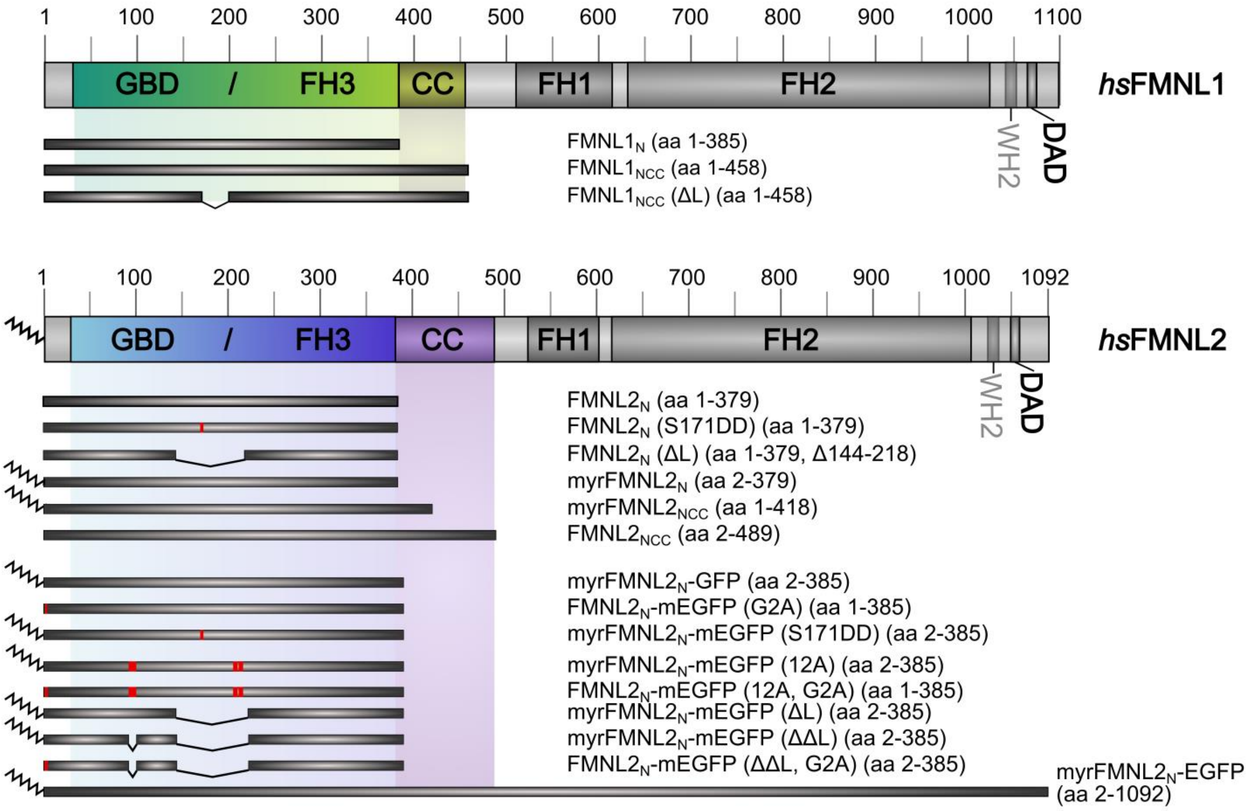

Supplementary Fig. 8 | Protein constructs of human FMNL1 and FMNL2 used in this study.

Modular domain architecture of human FMNL1 and FMNL2 proteins displayed as bar diagrams. For FMNL1, isoform 1 (UniProt accession number O95466) is shown and for FMNL2, isoform 2 (Q96PY5). Domain boundaries and construct designations are drawn below the diagrams. Mutant FMNL2 proteins used for the analysis of membrane binding properties in cells were fused at the C-terminus with mEGFP. For expression of recombinant FMNL2 proteins, myristoylation was achieved by co-expression of the N-myristoyl-transferase and addition of myristic acid.

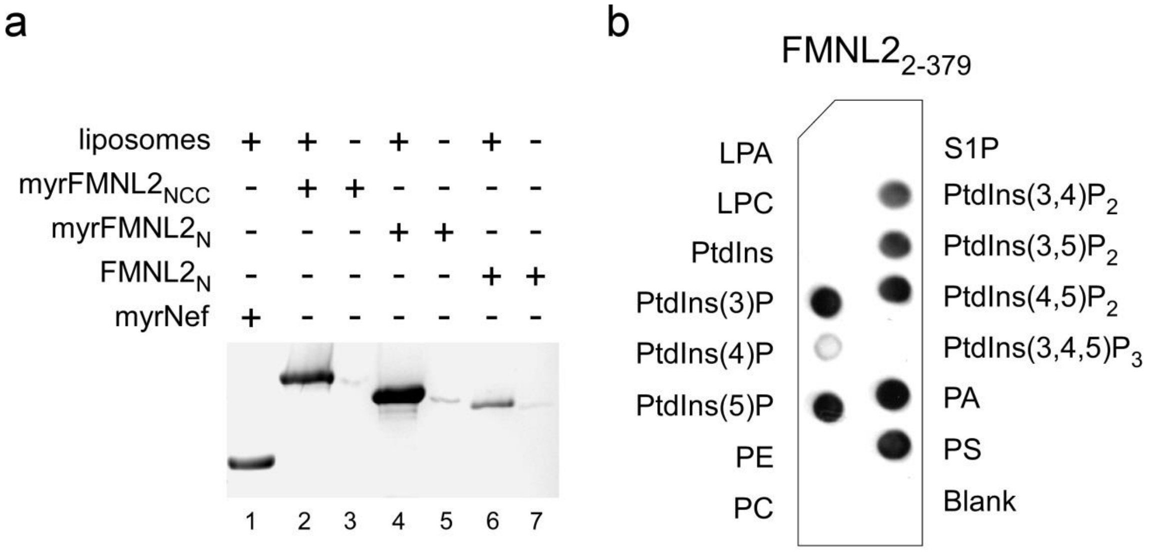

Supplementary Fig. 9 | Association of FMNL2 to liposomes and lipid components.
(**a**) Co-sedimentation assays of FMNL2 N-terminal domains to liposomes (DOPC/DOPG 70:30, r_H = 56 nm). Myristoylation of the formin protein significantly strengthens membrane association (compare lanes 2 and 4 to lane 6). As a control myristoylated HIV-1 Nef protein was used (lane 1). (**b**) Analysis of the association of $FMNL2_N$ binding to phosphoinositol-phosphates using a PIP strip assay. FMNL2 predominantly associates to negatively charged lipids whereas the non-charged lipids PC, PE and LPC are not bound. Overall, the PIP strip screen suggests that $FMNL2_N$ has no preference for a specific inositol phosphate head group but rather associates to negatively charged lipids in general. C-terminally His-tagged FMNL2 was detected by an anti-His antibody.

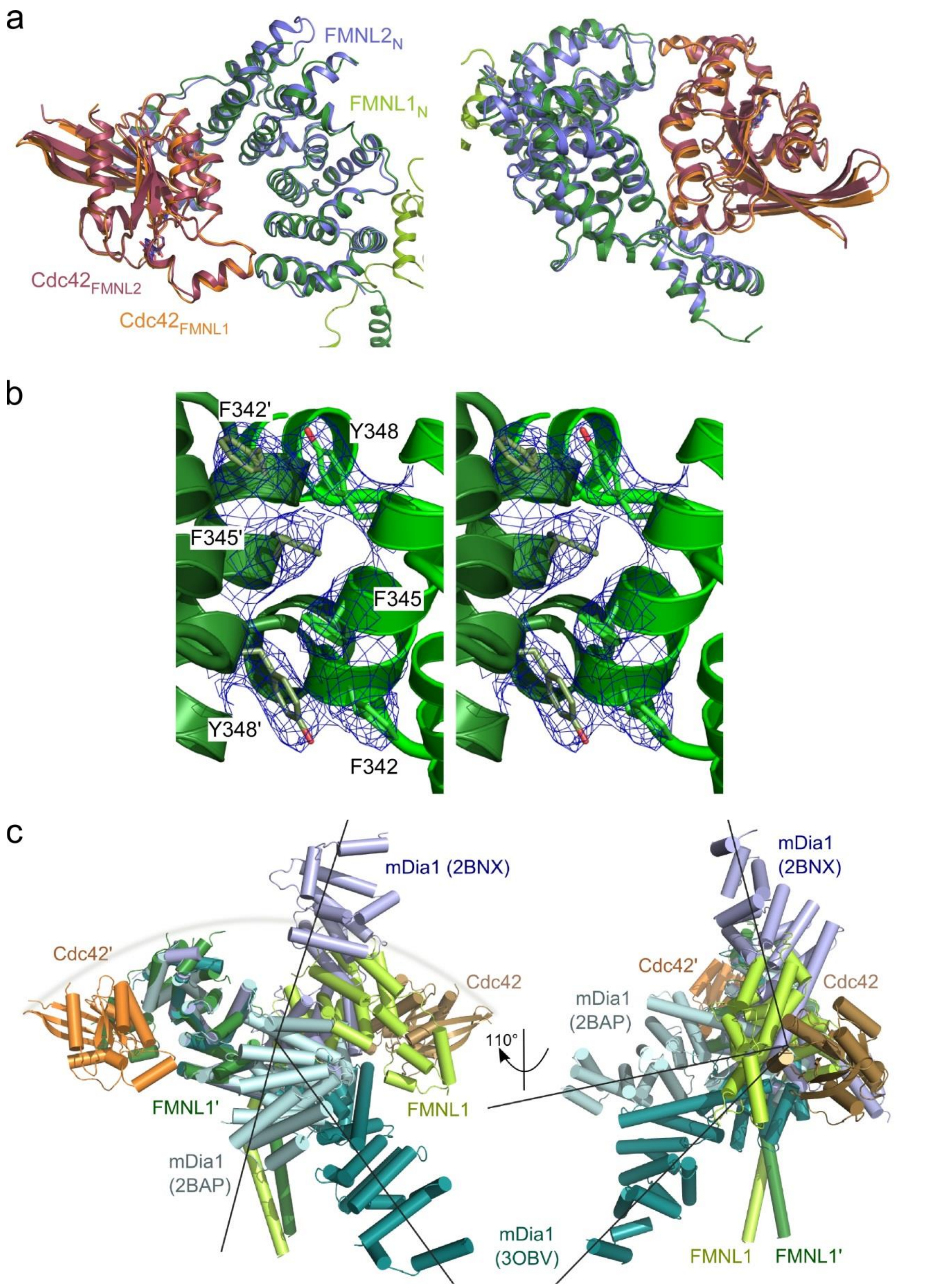

Supplementary Fig. 10 | Overlay of formin N-terminal domain structures.
(**a**) The complex structures of $FMNL1_N$/Cdc42 (4YDH) and $FMNL2_N$/Cdc42 (4YC7) share a high degree of similarity resulting in an RMSD value of 0.63 Å. (**b**) Stereo image of dimer interactions in FMNL1. The final $2F_o–F_c$ electron density is displayed at 1σ. (**c**) The structure assemblies of the three published mDia1 dimers 2BAP (ref. 4), 2BNX (ref. 5) and 3OBV (ref. 6) are significantly different from the symmetric dimer of FMNL1 (4YDH).

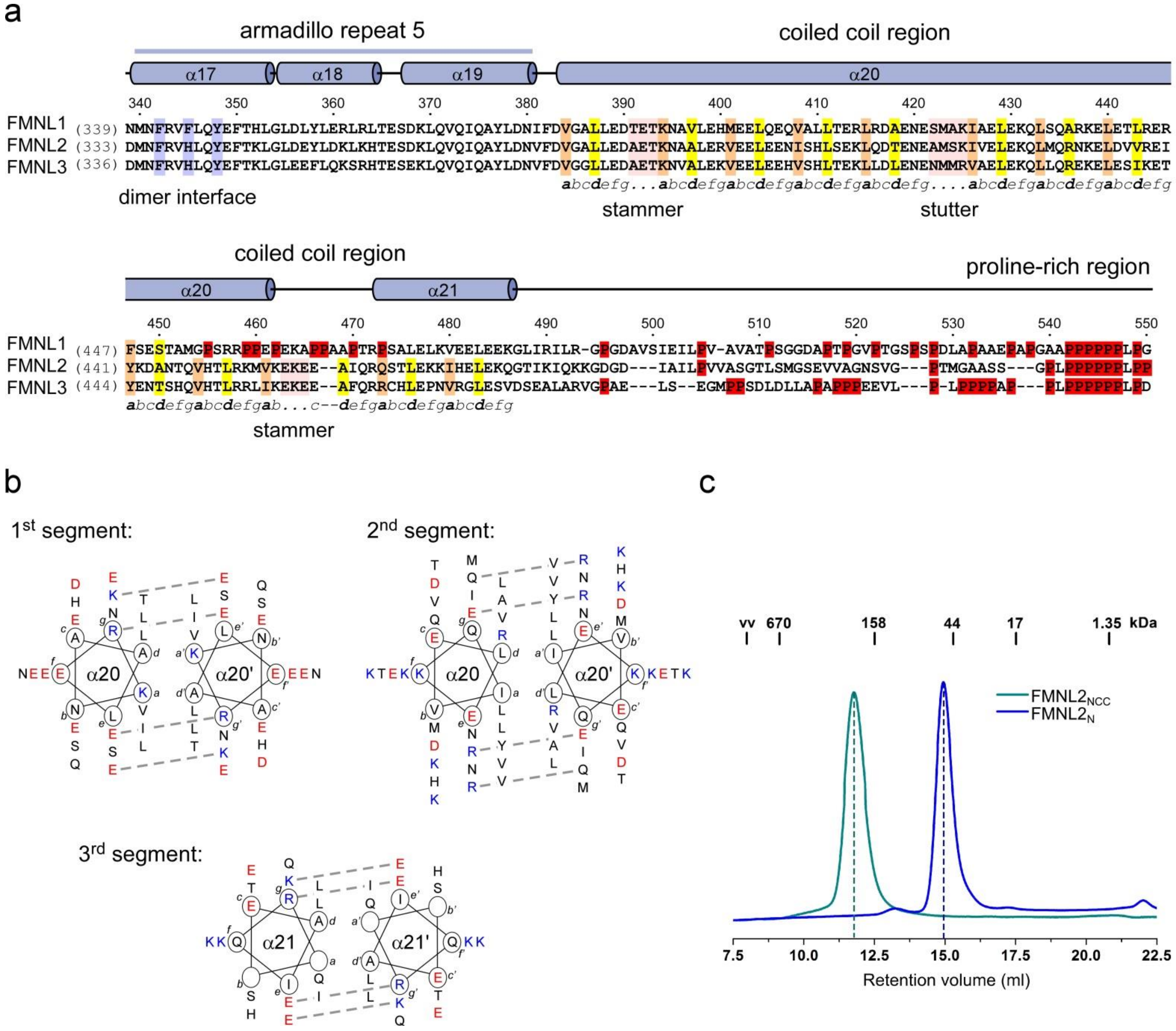

Supplementary Fig. 11 | Proposed coiled coil structures of FMNL formins.

(**a**) Sequence alignment of human FMNL formins. Secondary structure elements of FMNL1 are displayed on top of the sequence. Three aromatic residues in the first helix α17 of the fifth armadillo repeat contribute to homomeric dimer formation. The coiled coil region starts directly after the armadillo repeat containing FH3 domain at position 384 in FMNL1. The canonical hepta-repeat structure labeled as **a**bc**d**efg can be assigned for 10 repeats in FMNL1 and about 12 repeats in FMNL2 and FMNL3. The hepta-repeats are interrupted by 'stammer' and 'stutter' elements of 3 and 4 amino acid insertions, respectively, which contribute to the flexibility of the coiled coil domain. Proline residues indicating the beginning of the FH1 domain are boxed red. (**b**) Helical wheel display of the three coiled coil segments in FMNL2. The three elements arise by reason of the stutter and stammer elements. Positively charged amino acids are displayed in blue, negatively charged amino acids are displayed in red. Possible electrostatic interactions between the g_i position and the $e'_{(i+1)}$ position of the opposing strand that stabilize the coiled coil interaction and organize the register of the periodic heptad are indicated by dashed lines. (**c**) Addition of the coiled coil region to FMNL2 domain constructs leads to a significant increase in the apparent size of the protein. The early elution volume indicates formation of a protein dimer.

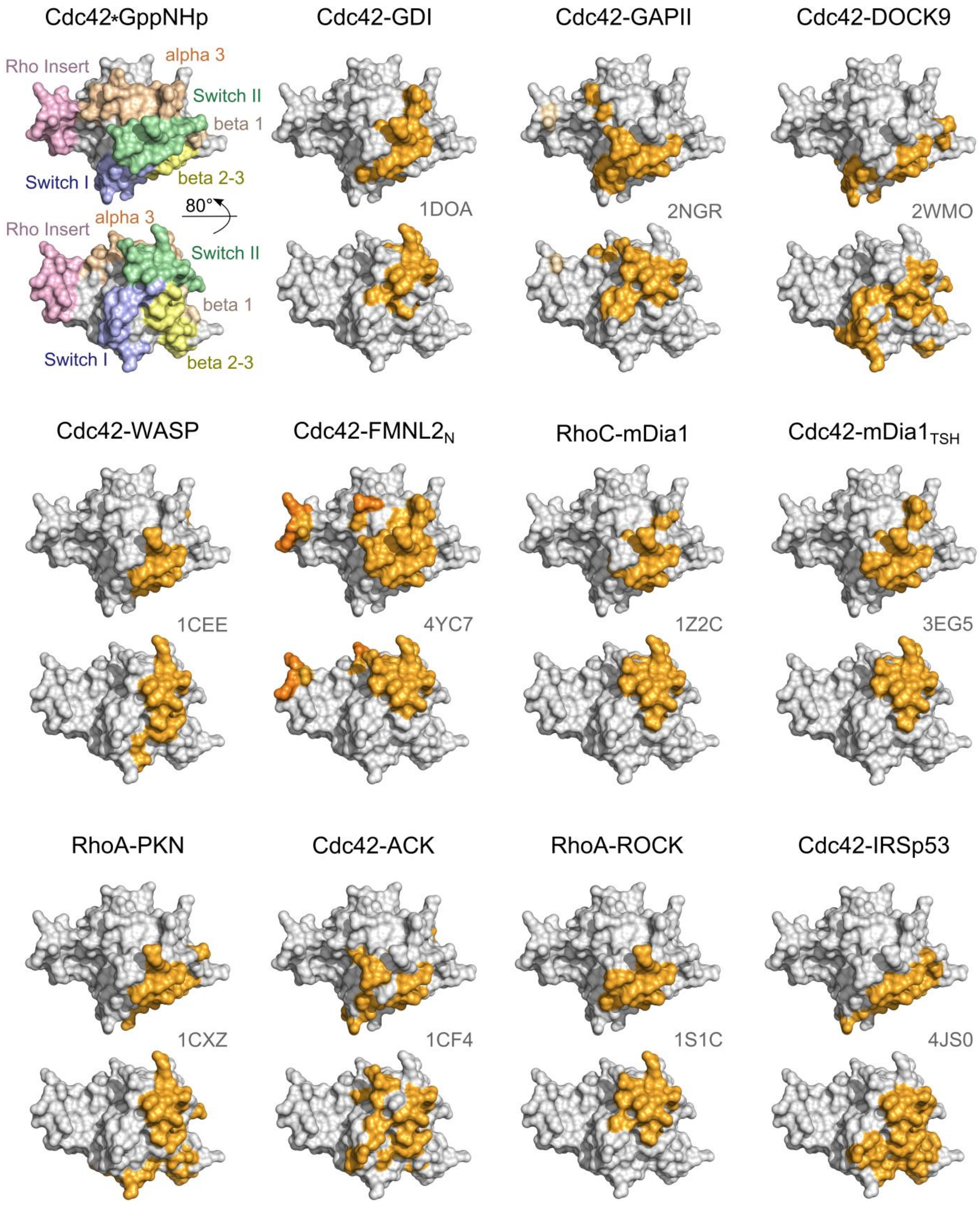

Supplementary Fig. 12 | Comparison of contact interfaces on Rho GTPases.
The interaction surfaces of various Rho GTPase-binding factors are projected on the structure of active Cdc42 displayed in two different views, each. The GTPase determining entities switch I and II, β-strands 1 and 2-3, α-helix 3 and the Rho GTPase specific insert helix α4 are shown. Surface interactions with guanine dissociation inhibitors, GTPase-activating factors, exchange factors, and effectors are displayed. The respective PDB codes are given in the figures.

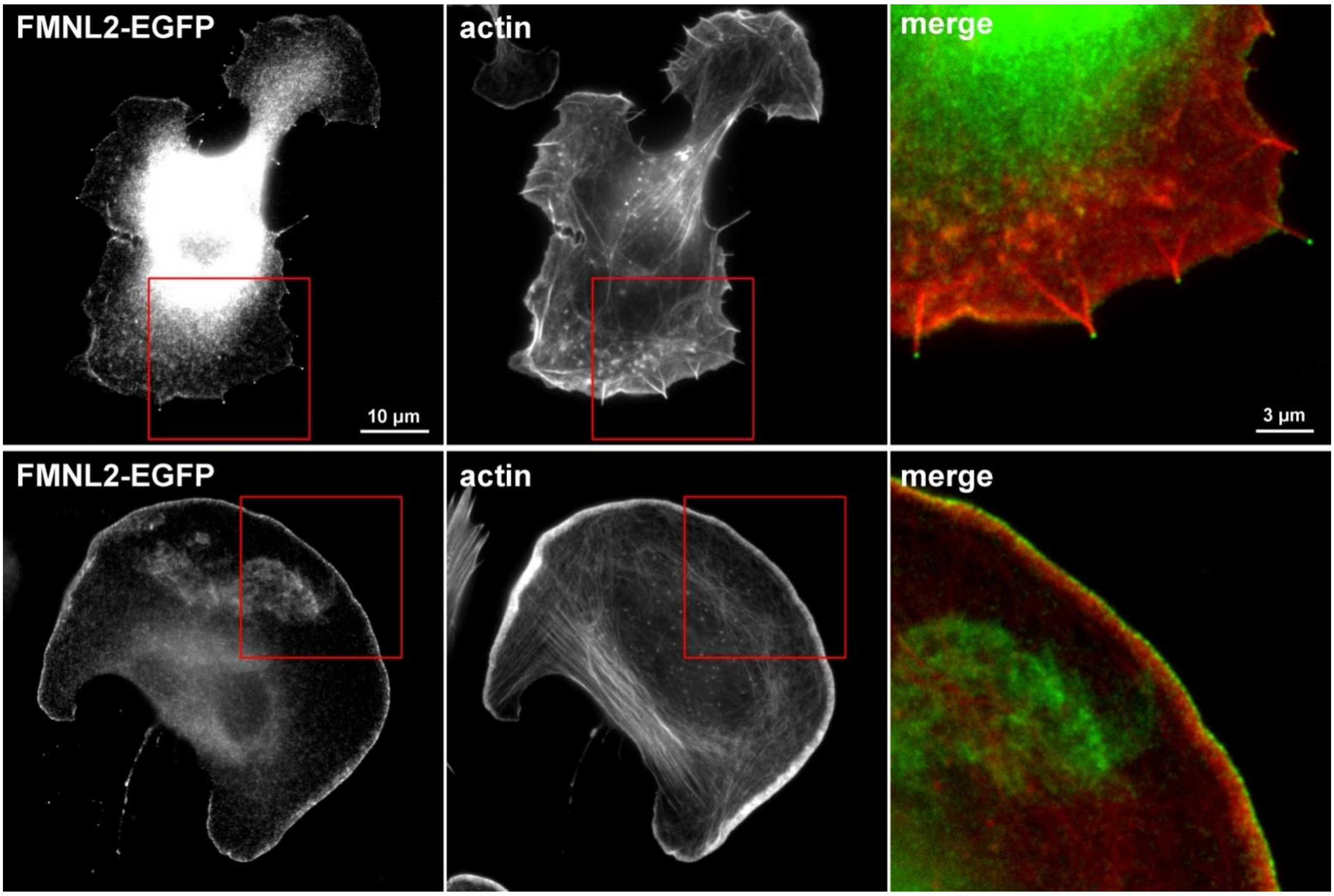

Supplementary Fig. 13 | Localization of FMNL2 in B16-F1 cells.
B16-F1 cells transiently expressing FMNL2-EGFP (left panels) were fixed and counterstained for the actin cytoskeleton using phalloidin (middle panels). Right panels show magnified insets (merge) boxed in overview images on middle and left panels. FMNL2 and actin filaments in merged panels are displayed in green and red, respectively. Note the accumulation of FMNL2 at the tips of filopodia and lamellipodia.

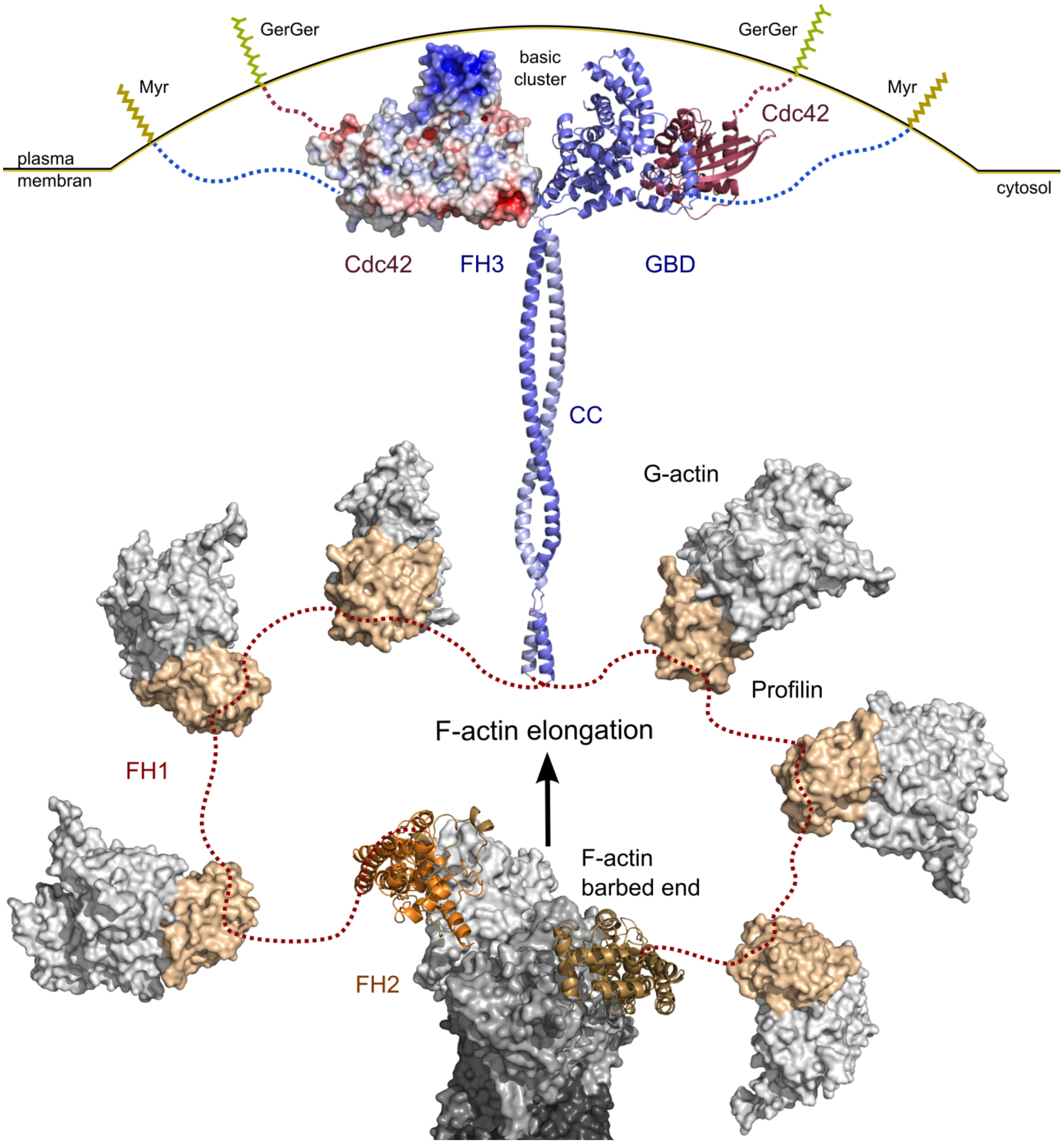

Supplementary Fig. 14 | Model of active, membrane bound FMNL2 upon actin elongation

Shown is a model of full length FMNL2 based on the crystal structures determined in this work and structural modeling. The Cdc42/FMNL2 dimer is attached to membranes by its lipidation motifs and the basic cluster. Actin filament elongation takes places by the stair-stepping mechanism of the FH2 domain with profilin–actin molecules being recruited by the prolin-rich FH1 domain. For structure display and structural modeling following PDB files were used: F-actin: 4A7F (ref. 7); poly-proline–profilin–actin: 2PBD (ref. 8) superimposed with 1ATN (ref. 9); FH2 (DAAM1): 2J1D (ref. 10); FH2–actin (FMNL3): 4EAH (ref. 11); FH2–actin (Bni1p): 1Y64 (ref. 12); FH1–profilin: 2V8F (ref. 13). Abbreviations correspond to FH1/FH2/FH3: Formin homology domain 1/2/3; CC: coiled coil; GBD: GTPase binding domain; Myr: myristoylation; GerGer: geranylgeranylation.

Supplementary References

1. Rojas, A.M., Fuentes, G., Rausell, A. & Valencia, A. The Ras protein superfamily: evolutionary tree and role of conserved amino acids. *J. Cell. Biol.* **196**, 189–201 (2012).
2. Lammers, M., Meyer, S., Kühlmann, D. & Wittinghofer, A. Specificity of interactions between mDia isoforms and Rho proteins. *J. Biol. Chem.* **283**, 35236–35246 (2008).
3. Abdul-Manan N, Aghazadeh B, Liu GA, Majumdar A, Ouerfelli O, Siminovitch KA, Rosen MK. Structure of Cdc42 in complex with the GTPase-binding domain of the 'Wiskott-Aldrich syndrome' protein. *Nature* **399**, 379–383 (1999).
4. Lammers, M., Rose, R., Scrima, A. & Wittinghofer, A. The regulation of mDia1 by autoinhibition and its release by Rho*GTP. *EMBO J.* **24**, 4176–4187 (2005).
5. Otomo, T., Otomo, C., Tomchick, D.R., Machius, M. & Rosen, M.K. Structural basis of Rho GTPase-mediated activation of the formin mDia1. *Mol. Cell* **18**, 273–281 (2005).
6. Otomo, T., Tomchick, D.R., Otomo, C., Machius, M. & Rosen, M.K. Crystal structure of the Formin mDia1 in autoinhibited conformation. *PLoS One* **5**, e12896 (2010).
7. Behrmann, E., Müller, M., Penczek, P.A., Mannherz, H.G., Manstein, D.J. & Raunser, S. Structure of the rigor actin-tropomyosin-myosin complex. *Cell* **150**, 327–338 (2012).
8. Ferron, F., Rebowski, G., Lee, S.H. & Dominguez, R. Structural basis for the recruitment of profilin-actin complexes during filament elongation by Ena/VASP. *EMBO J.* **26**, 4597–4606 (2007).
9. Kabsch, W., Mannherz, H.G., Suck, D., Pai, E.F. & Holmes, K.C. Atomic structure of the actin:DNase I complex. *Nature* **347**, 37-44 (1990).
10. Lu, J., Meng, W., Poy, F., Maiti, S., Goode, B.L. & Eck, M.J. Structure of the FH2 domain of Daam1: implications for formin regulation of actin assembly. *J. Mol. Biol.* **369**, 1258–1269 (2007).
11. Thompson, M.E., Heimsath, E.G., Gauvin, T.J., Higgs, H.N. & Kull, F.J. FMNL3 FH2-actin structure gives insight into formin-mediated actin nucleation and elongation. *Nat. Struct. Mol. Biol.* **20**, 111–118 (2013).
12. Otomo, T., Tomchick, D.R., Otomo, C., Panchal, S.C., Machius, M. & Rosen, M.K. Structural basis of actin filament nucleation and processive capping by a formin homology 2 domain. *Nature* **433**, 488–494 (2005).
13. Kursula, P., Kursula, I., Massimi, M., Song, Y.H., Downer, J., Stanley, W.A., Witke, W. & Wilmanns, M. High-resolution structural analysis of mammalian profilin 2a complex formation with two physiological ligands: the formin homology 1 domain of mDia1 and the proline-rich domain of VASP. *J. Mol. Biol.* **375**, 270–290 (2008).
